# “Drug‐Carrier” Synergy Therapy for Amyloid‐*β* Clearance and Inhibition of Tau Phosphorylation via Biomimetic Lipid Nanocomposite Assembly

**DOI:** 10.1002/advs.202106072

**Published:** 2022-03-20

**Authors:** Guochen Han, Kaiwen Bai, Xiaoyu Yang, Chenhua Sun, Yi Ji, Jianping Zhou, Huaqing Zhang, Yang Ding

**Affiliations:** ^1^ Key Laboratory of Drug Quality Control and Pharmacovigilance (Ministry of Education) State Key Laboratory of Natural Medicines Department of Pharmaceutics China Pharmaceutical University Nanjing 210009 China

**Keywords:** Alzheimer's disease, carrier for A*β* targeting and clearance, “Drug‐Carrier” synergy treatment, high BBB penetration, methylene blue inhibiting Tau phosphorylation

## Abstract

Amyloid‐*β* (A*β*) toxicity is considered to be companioned by Tau phosphorylation in Alzheimer's disease (AD). The clinical AD therapy is usually subjected to low blood‐brain barrier (BBB) penetration and complex interaction mechanisms between A*β* and phosphorylated Tau. A “Drug‐Carrier” synergy therapy is herein designed to simultaneously target A*β* and Tau‐associated pathways for AD treatment. To imitate natural nanoparticle configuration, the endogenous apolipoprotein A‐I and its mimicking peptide 4F fused angiopep‐2 (Ang) are sequentially grafted onto lipid nanocomposite (APLN), providing liberty of BBB crossing and microglia targeted A*β* clearance. For synergy treatment, methylene blue (MB) is further assembled into APLN (APLN/MB) for Tau aggregation inhibition. After intravenous administration, the optimized density (5 wt%) of Ang ligands dramatically enhances APLN/MB intracerebral shuttling and accumulation, which is 2.15‐fold higher than that Ang absent‐modification. The site‐specific release of MB collaborates APLN to promote A*β* capture for microglia endocytosis clearance and reduce p‐Tau level by 25.31% in AD pathogenesis. In AD‐A*β*–Tau bearing mouse models, APLN/MB can relieve AD symptoms, rescue neuron viability and cognitive functions. Collectively, it is confirmed that “Drug‐Carrier” synergy therapy of APLN/MB is a promising approach in the development of AD treatments.

## Graphical abstract

Clinical Alzheimer's disease (AD) treatment faces dilemmas in low blood‐brain barrier (BBB) penetration and complex interaction mechanisms between A*β* deposits and phosphorylated Tau. This study provides a biomimetic lipid nanocomposite (APLN/methylene blue) with high BBB penetration for AD therapy via a “Drug‐Carrier” synergy strategy, of which carrier for A*β* clearance and drug for inhibition of phosphorylated Tau.

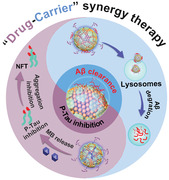

## 1 Introduction

Alzheimer's disease (AD) is one of neurodegenerative diseases, characterized by extracellular deposition of amyloid‐*β* (A*β*) plaques and intracellular neurofibrillary tangles (NFTs) of hyperphosphorylated Tau protein.^[^ ^1^ ^]^ More than 50 million global patients are suffering from AD and the clinical AD treatment is always compromised of symptom relief by treating with donepezil, tacrine, galantamine, memantine, and rivastigmine.^[^ ^2^ ^]^ Even worse, the failure rate of clinical trials was approaching 100% because of complex pathological mechanisms of AD and low drug penetration of blood‐brain barrier (BBB).^[^ ^3^ ^]^ Therefore, the development of novel medicine therapeutic strategies is critically needed.

The prevalent views of AD pathogenesis include the amyloid hypothesis and the Tau hypothesis. However, the underlying mechanisms and A*β*–Tau interrelationships are still enigmatic. Currently, A*β* accumulation indicates the initial cause that drives Tau accumulation and Tau‐mediated neurotoxicity in AD.^[^ ^4^ ^]^ Anti‐A*β* antibody and nanoparticles‐mediated A*β* clearance have been the mainstream direction of anti‐AD drug development.^[^ ^5^ ^]^ Unfortunately, the compelling experimental and clinical data suggest that almost all efforts focused on clearing A*β* failed.^[^ ^6^ ^]^ Considering that A*β*‐initiated secondary pathological processes, especially Tau hyperphosphorylation could independently lead to neuronal degeneration and pathogenesis in AD, A*β* clearance alone is difficult to control AD progression.^[^ ^7^ ^]^ These studies emerged that the copathogenic interaction between A*β* and Tau not only elucidated the failure of targeting A*β* alone, but also informed the next generation of research direction. Therefore, we confirm that A*β*–Tau synergy treatment that combined A*β* clearance and Tau inhibition simultaneously may be the most suitable way to combat the complex pathogenesis of AD.

For synergy treatment, the hindrance of Tau phosphorylation and aggregation is one of significant steps. As a microtubule‐associated protein, Tau functions as a stabilization of microtubules in neurons and plays a vital role in neurogenesis and axonal transport.^[^ ^8^ ^]^ Once Tau is hyperphosphorylated, protein aggregation cascade will be initiated, ultimately leading to insoluble NFTs.^[^ ^9^ ^]^ Accordingly, the targeted Tau treatment should focus on inhibiting Tau phosphorylation and aggregation in AD treatment.^[^ ^10^ ^]^ Methylene blue (MB) has been proved to share the potential of downregulation of the p‐Tau level and inhibiting Tau protein aggregation.^[^ ^11^ ^]^ However, the low efficiency of intracerebral accumulation, and short half‐life of MB are immediate downsides for application in AD therapy.^[^ ^12^ ^]^

The clearance of overexpressed A*β* is another critical strategy for synergy therapy, which could alleviate A*β*‐mediated pathology.^[^ ^13^ ^]^ Apolipoprotein A‐I (apoA‐I) as a functional component of natural lipid nanoparticles, is competent to capture and eliminate A*β*.^[^ ^14^ ^]^ Moreover, apoA‐I deficiency tends to accelerate cognitive decline in AD progress, while increased apoA‐I level will improve cognitive function and alleviate neuroinflammation through multiple mechanisms.^[^ ^15^ ^]^ As a result, apoA‐I‐enriched nanocarriers possess tremendous potential for AD treatment.^[^ ^16^ ^]^ The structure of the amphipathic *α*‐helical repeats promotes apoA‐I to insert into the lipid layer and form a protein–lipid nanocomposite (PLN).^[^ ^17^ ^]^ The biomimetic PLN can perform as a drug carrier for multidrug shielding, and maintain the high binding affinity to A*β*. To enhance intracranial drug delivery, a 19‐amino acid ligand of Angiopep‐2 (Ang) has been introduced to modify nanoparticle for high‐efficiency of BBB‐mediated lipoprotein receptor‐related protein 1 (LRP1) targeting and penetrating.^[^ ^18^ ^]^ Technically, Ang is usually conjugated with amphipathic peptides, polymers or phospholipids for nanoparticle surface modification.^[^ ^19^ ^]^

**Scheme 1:**
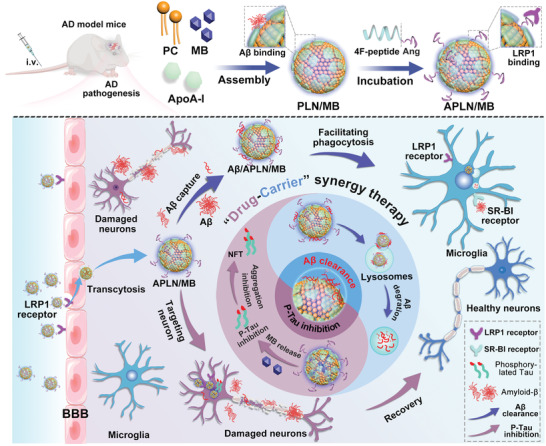
Scheme diagram of APLN/MB preparation and mechanism of “Drug‐Carrier” synergy therapy in AD mice. APLN/MB sequentially performs “Carrier” function for BBB crossing to remove A*β*, and implement site‐specific “Drug” release for ameliorating Tau‐related pathology.

Hence, we herein propose a biomimetic protein–lipid nanocomposite of APLN/MB for “Drug‐Carrier” synergy treatment, which can simultaneously target A*β* and Tau‐associated pathways. As illustrated in **Scheme** **1**, PLN is prepared through the bioinspired assembly of phosphatidylcholine and apoA‐I, and sequentially MB is encapsulated into the hydrophilic core. Afterwards, apoA‐I mimicking peptide of 4F (NH_2_‐FAEKFKEAVKDYFAKFWD‐COOH) is further fused with the optimized density of Ang ligand to modify nanoparticle of PLN/MB and improve brain targetability. In A*β*–Tau copathogenic mouse models, APLN/MB could perform “Drug‐Carrier” synergy therapy in AD lesions. As expected, APLN shares a higher A*β* binding affinity to facilitate A*β* capture and clearance mediated by microglia endocytosis. Due to the assistance of APLN carrier, MB is internalized into neuronal cells to inhibit intracellular phosphorylation and aggregation of Tau. Overall, APLN/MB nanoplatform with high BBB penetration can achieve the synergy therapy by targeting A*β* and Tau pathways, which provides deep insights into clinical AD therapy.

## 2 Results and Discussion

### 2.1 Preparation and Characterization of APLN/MB

**Figure 1:**
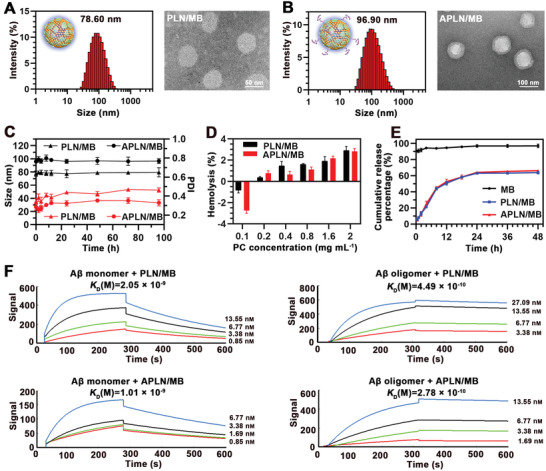
Characterization of preparations. A) Particle size and TEM image of PLN/MB. Scale bars = 50 nm. B) Particle size and TEM image of APLN/MB. Scale bars = 100 nm. C) Changes of particle size and polydispersity index (PDI) of PLN/MB and APLN/MB in PBS with 10% serum, respectively (*n* = 3). D) Hemolysis of PLN/MB and APLN/MB at series of lipid concentrations, respectively (*n* = 5). E) The percentages of released MB from PLN/MB and APLN/MB in PBS, respectively (*n* = 3). F) The binding affinity of PLN/MB and APLN/MB to A*β*_1‐42_ monomer and oligomer was measured by SPR, respectively. Data were presented as mean ± SD.

The natural particulate of high density lipoprotein (HDL) represents the ideal drug delivery carrier for effective intracerebral drug delivery and A*β* clearance in AD treatment.^[^ ^20^ ^]^ As the most important functional component of HDL, apolipoprotein A‐I (apoA‐I) plays a crucial role in BBB penetration and A*β* clearance.^[^ ^21^ ^]^ However, the hydrophobic core of the lipid monolayer structure of natural particulate is not applicable for the delivery of the hydrophilic drug. Hence, natural biomimetic PLN composed of apoA‐I and phosphatidylcholine has been designed to mimic the function and structure of HDL for brain delivery of hydrophilic MB. MB‐loaded protein and lipids nanocomposite (PLN/MB) was prepared by using the thin‐film dispersion and lipid film hydration method. As determined by dynamic light scattering (DLS) and visualized by transmission electron microscopy (TEM), PLN/MB shared the particle size of 78.60 nm and homogenous spherical morphology with the zeta potential of −28.64 mV (**Figure** **1**A; Table S1, Supporting Information).

To further improve the intracerebral accumulation, a 19‐mer peptide of Ang was employed for binding low‐density LRP1, which is a well‐documented receptor for BBB crossing. In the biogenesis of native HDL, apoA‐I can bind and embed into lipids due to the amphiphilic property of *α*‐helical repeats, leading to the formation of HDL.^[^ ^22^ ^]^ To stimulate this bioprocess, the apoA‐I mimic peptide 4F was developed with repeated *α*‐helical repeats and fused with Ang (*α*Ang). Different from traditional chemical modification, *α*Ang could be anchored onto the lipid surface of PLN/MB through the amphiphilic property of 4F. The dynamic binding behavior of peptide with the lipid layer was monitored by surface plasma resonance (SPR) analysis.^[^ ^23^ ^]^ As shown in Figure S1 of the Supporting Information, 4F and *α*Ang peptide displayed similar binding behavior to the surface of lipid with the binding affinity constant (*K*_D_) values of 2.48 × 10^−6^ and 1.58 × 10^−6^ m, respectively. By contrast, ignorable signal was detected when the Ang peptide flow through the sensor chip, indicating the undetectable binding affinity between Ang and the lipid surface. The results demonstrated that *α*Ang could anchor onto the lipid surface via a 4F‐mediated biomimic manner of modification.^[^ ^24^ ^]^ After surface decoration with *α*Ang, the hydrodynamic diameter of APLN/MB nanoparticles increased to 96.90 nm, which could be observed in TEM image and DLS (Figure 1B). In addition, PLN/MB and APLN/MB displayed MB entrapment efficiency of (70.53 ± 0.95)% and (71.88 ± 1.30)%, and drug loading of (2.50 ± 0.07)% and (2.44 ± 0.03)%, respectively (Table S1, Supporting Information), which were quantified by UV–vis measurements. The results suggested that the protein‐lipid structure of PLN could provide enough hydrophilic core for MB encapsulation.

When incubated in PBS with 10% serum, no obvious size variation could be observed in both groups of APLN/MB and PLN/MB in 96 h, indicating desirable serum stability for further application (Figure 1C). Moreover, APLN/MB and PLN/MB displayed no negligible hemolytic activity (<4%, Figure 1D), providing the guarantee of safety for intravenous administration. To investigate the controllable drug release, MB release profiles from different preparations were analyzed with the release medium of PBS (pH 7.4). Free MB was almost completely released in 0.5 h, indicating no diffusion hindrance by the dialysis bag. Compared to free MB, PLN/MB exhibited controllable drug release that reached ≈60% in 24 h with no burst initial release (Figure 1E). Similar release patterns were observed in APLN/MB, demonstrating that the decoration of *α*Ang ligands did not affect the release behavior of MB obviously.

### 2.2 High Binding Affinity to A*β* Monomer and Oligomer

The recognition and binding affinity of A*β* are indispensable for its clearance and determine the clearance efficacy. As reported, apoA‐I possessed high A*β*‐binding affinity for enhanced A*β* clearance.^[^ ^20^ ^]^ Hence, the protein and lipid nanocomposite with apoA‐I could inherit A*β* binding affinity from native HDL. To validate this assumption, we evaluated and compared the binding affinity of PLN/MB and APLN/MB to A*β* monomer and oligomer by SPR analysis, respectively.^[^ ^25^ ^]^ A*β*_1‐42_ monomer or oligomer was connected via an amine coupling reaction on the surface of the sensor. The binding affinity constant (*K*_D_) was obtained by using a 1:1 Langmuir binding model. The *K*_D_ values of PLN/MB to A*β*_1‐42_ monomer and oligomer were 2.05 × 10^−9^ m and 4.49 × 10^−10^ m, respectively (Figure 1F; Table S2, Supporting Information). The *K*_D_ values of APLN/MB to A*β*_1‐42_ monomer and A*β*_1‐42_ oligomer were reduced by 49.27% and 61.92% than PLN/MB, respectively, indicating that *α*Ang modification could maintain and even improve A*β*‐binding affinity. Considering that the 4F peptide could mimic apoA‐I, it was not surprising to see that *α*Ang could provide additional binding response to A*β* with improved binding affinity. This evidence suggested that the A*β*‐binding affinity of PLN/MB could be elevated by the specific synergy effect of apoA‐I and 4F. In addition, the *K*_D_ values of APLN/MB to A*β*_1‐42_ oligomer were lower than that of A*β*_1‐42_ monomer, which suggested that APLN/MB provided a higher binding affinity to A*β*_1‐42_ oligomer. As the most toxic form of A*β*, A*β*_1‐42_ oligomer can trigger neuronal apoptosis by attacking the neuron membranes. The higher binding affinity to A*β*_1‐42_ oligomer could help APLN/MB selectively capture A*β* oligomer for clearance, thus protecting neuron cells from A*β* oligomer induced cell damage.

### 2.3 Establishment and Evaluation of AD Model Mice of Dual‐Etiology

Current studies indicated that the complex A*β*–Tau interaction synergistically drove AD progression.^[^ ^26^ ^]^ To evaluate the therapeutic effects of “Drug‐Carrier” synergy of APLN/MB, an animal model is required to simulate the A*β*–Tau pathology and other hallmarks of AD. Compared with the transgenic mice model, the intracerebral administration of AD pathogenic substances gives the advantages of low cost, simple operation and is suitable for a large‐scale laboratory study.^[^ ^27^ ^]^ Moreover, this AD mouse model shows the relatively reproducibility and homogeneous degree of Tau or A*β* pathology in a short period, respectively. Nevertheless, there is no report regarding available about A*β*–Tau copathogenic AD mice models through intracerebral administration of dual‐toxics simultaneously. As a result, a new AD mouse model was proposed and established through bilateral intrahippocampal coinjection of A*β*_1‐42_ (82 µmol L^−1^) and okadaic acid (OA) with different concentrations (low dose of 1 µmol L^−1^, middle dose of 2 µmol L^−1^, and high dose of 4 µmol L^−1^), named as OA (L) + A*β* group, OA (M) + A*β* group and OA (H) + A*β* group, respectively. Mice with bilateral intrahippocampal coinjection of saline, A*β*_1‐42_ (82 µmol L^−1^) or OA (2 µmol L^−1^) were used as control and denoted as Sham, A*β* and OA, respectively.

**Figure 2:**
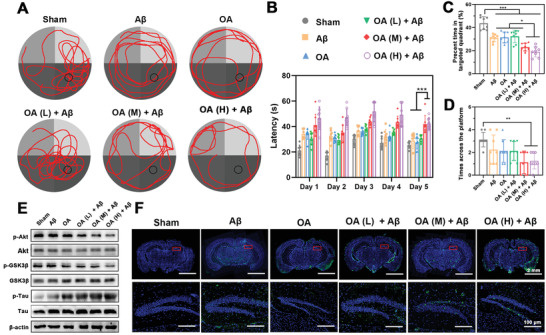
Evaluation of cognitive, memory impairments, and A*β*–Tau pathological hallmarks of AD model mice. A) The representative swimming paths of mice in the MWM test. B) Latency (*n* = 8). C) Percent time spent on the targeted quadrant (*n* = 8). D) Times across the platform (*n* = 8). E) Western blot results of p‐Akt, Akt, p‐GSK3*β*, GSK3*β*, p‐Tau, and Tau in mice brain. F) Immunostaining of A*β* in the whole brain and representative of DG region of the hippocampus. Scale bars = 2 mm or 100 µm. Data were presented as mean ± SD. *p < 0.05, **p < 0.01, and ***p < 0.001.

The cognitive and memory impairments, and pathological hallmarks of A*β* deposition and Tau hyperphosphorylation are key indexes to evaluate the copathogenic AD mice models. Hence, Morris Water Maze (MWM) and nesting behavior were performed to evaluate memory and cognitive deficits of AD model mice. In MWM test, mice in Sham group showed an aim searching strategy (**Figure** **2**A) and short escape latency time of 20 s (Figure 2B). Compared with the mice in Sham group, mice in A*β*, OA, and OA (L) +A*β* groups were similar in terms of escape latency time. By contrast, the latency time of mice in OA (M) + A*β* and OA (H) + A*β* groups were significantly increased (≈40 and ≈45 s, respectively), indicating that the spatial learning and memory were impaired by A*β* and OA induced p‐Tau pathology. Similarly, when the platform was removed, mice in OA (M) + A*β* and OA (H) + A*β* groups showed poorly platform searching performance with a lower percent time in the targeted quadrant (≈21% and ≈18%, respectively) and fewer times across the platform (Figure 2C,D). Moreover, the nesting behavior was performed to assess the hippocampal function. As shown in Figure S2 of the Supporting Information, the Sham group mice could build a complete and mass nest, while the nested score was slightly decreased after bilateral intrahippocampal injection of A*β*_1‐42_ and OA alone, respectively. Notably, the nesting score of mice in OA (M) + A*β* and OA (H) + A*β* groups were significantly decreased compared with that of Sham group due to the cognitive decline deficiency. The results demonstrated that the bilateral intrahippocampal coinjection of A*β*_1‐42_ with OA (2 and 4 µmol L^−1^) could produce neuronal damage and loss, induce learning and memory deficits.

To investigate pathologic consequences of A*β*‐OA cointeractions in AD model mice, western blot and immunofluorescence analysis were further performed to evaluate Tau phosphorylation and A*β* deposition, respectively. Akt/GSK3*β* signaling pathway is crucial in Tau pathogenesis.^[^ ^28^ ^]^ Tau kinase of glycogen synthase kinase3*β* (GSK3*β*) promotes Tau phosphorylation into p‐Tau. When phosphorylated by phosphorylated protein kinase B (p‐Akt), GSK3*β* would transform into an inactive state (p‐GSK3*β*). To illustrate whether OA could inactive the Akt/GSK3*β* signaling pathway to induce Tau pathogenesis in mice, the expression of p‐Akt, p‐GSK3*β*, and p‐Tau in mice brains were detected by western blot, respectively. As shown in Figure 2E and Figure S3 (Supporting Information), the highest p‐Tau expression was observed in the OA (M) + A*β* group, which was nearly the same as that of OA (H) + A*β* group, indicating that A*β* together with both middle and high dose of OA could significantly induce the overexpression of p‐Tau. It is noteworthy that the p‐Tau level was also increased in A*β* group, which proved that A*β* could drive Tau pathology in AD model mice. Moreover, we further analyzed A*β* deposition by immunofluorescence in the brain of model mice. As expected, massive A*β* deposition was observed in brains of the A*β* group mice (Figure 2F), while the mice coinjected with A*β*_1‐42_ and OA emerged increased A*β* deposition in the dentate gyrus (DG) region of hippocampus with the OA concentration increase. The results indicated that OA could promote A*β* pathological progress. Surprisingly, there were some A*β* depositions in the OA group, revealing that phosphorylated Tau induced by OA could ultimately lead to the deposition of A*β*. These results indicated that both bilateral intrahippocampal coinjection of OA (M) + A*β* or OA (H) + A*β* were able to present A*β* and Tau pathology in the brain, which could provide an effective mice model for studying A*β*–Tau synergy therapy. Considering the similar effect in cognitive impairments and pathological hallmarks, bilateral intrahippocampal coinjection of OA (M) + A*β* was chosen as the optimal method for establishing A*β*–Tau copathological mice model and evaluating “Drug‐Carrier” synergy therapy of APLN/MB subsequently.

### 2.4 Optimization and Analysis of BBB Penetration

**Figure 3:**
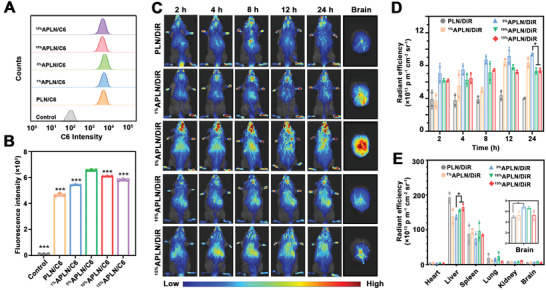
Analysis of BBB penetration both in vitro and in vivo. A) Flow cytometric analysis of hCMEC/D3 cellular uptake after treated with different formulations for 4 h (*n* = 3). B) The mean C6 fluorescence intensity of hCMEC/D3 cells treated with various nanoparticles. Compared with the 5% APLN/C6 group (*n* = 3). C) In vivo imaging of mice administrated with PLN/DiR with various *α*Ang densities of 1%, 5%, 10%, and 15% and ex vivo imaging of brains in different groups after 24 h. D) Quantitative assessment of in vivo fluorescence intensity of brains at different time intervals (*n* = 3). E) The quantitative fluorescence intensity of major organs (*n* = 3). Data were presented as Mean ± SD. *p < 0.05 and ***p < 0.001.

Clinicopathological studies have observed high expression levels of LRP1 in human brain endothelium, neurons, and activated microglia in the brain of AD patients.^[^ ^29^ ^]^ Moreover, Ang peptide has been evaluated in clinical trials for LRP1‐mediated BBB crossing via noninvasive transcytosis.^[^ ^30^ ^]^ Efficient transcytosis requires enough ligands for the recognition and binding with receptors for effectively trafficked across BBB. However, the higher density of ligand could not only decrease the binding ability of nanoparticles to receptors due to steric hindrances caused by too closely adjacent ligands, but also possibly enhance nonspecific interactions in vivo.^[^ ^31^ ^]^ Therefore, an optimal ligand density is required to promote sufficient BBB crossing. To optimize the efficiency of BBB penetration, preparations with different *α*Ang ligand densities of 1%, 5%, 10%, and 15% (corresponding lipids were denoted as ^1%^APLN/MB, ^5%^APLN/MB, ^10%^APLN/MB, and ^15%^APLN/MB, respectively) were prepared. MB was replaced by fluorescent dyes of C6 for nanoparticle tracking. The preparations were incubated with hCMEC/D3 cells with fluorescence intensity of C6 measured. Flow cytometric analysis showed the uptake of ^1%^APLN/C6 by hCMEC/D3 cells was 1.27‐fold higher than that of PLN/C6, suggesting that *α*Ang modification could enhance the cellular uptake (**Figure** **3**A,B). By contrast, the C6 fluorescence intensity of cells treated with ^5%^APLN/MB was 1.51‐fold higher than that of ^1%^APLN/MB. However, the C6 fluorescence intensity was decreased in cells when treated with ^10%^APLN/C6 and ^15%^APLN/C6, indicating that the cellular uptake of APLN/C6 was decreased with *α*Ang density further increased. The decreased cellular uptake was due to that the excessive density of *α*Ang with steric hindrances would block the binding with LRP1 receptor.

Given the complex physiological microenvironment of BBB, the optimal *α*Ang ligand density (5%) screened at the cytological level was not completely convincing. To investigate BBB transcytosis ability, biodistribution and accumulation of nanoparticles, the near‐infrared probe DiR was loaded in various nanocomposites (including PLN/DiR, ^1%^APLN/DiR, ^5%^APLN/DiR, ^10%^APLN/DiR, and ^15%^APLN/DiR), and intravenously injected in A*β*–Tau dual‐etiology AD mice. As shown in Figure 3C, more brain accumulation of ^5%^APLN/DiR was found in AD mice and the fluorescence intensity collected in the brain was 2.15‐fold higher than PLN/DiR at 24 h (Figure 3D), suggesting that Ang modification can help cargos cross BBB and enhance the distribution in the brain. Interestingly, when *α*Ang ligand density increased from 5% to 10% or 15%, the accumulation of APLN/DiR in the brains was not elevated but declined. This result might be attributed to the excessive density of Ang that would lead to the decreased BBB penetration capability of nanoparticles with crowding effect. In addition, the LRP1 receptors also express on the peripheral organs, such as the liver and heart, resulting in the increased liver accumulation in groups of ^10%^APLN/DiR and ^15%^APLN/DiR (Figure 3E). On the other hand, the average radiant efficiency of major organs in the ^5%^APLN/DiR group was lower than that of other groups, suggesting that the optimal density modification of *α*Ang‐peptide might reduce organ accumulation and improve the brain targeting of nanocomposites. Hence, ^5%^APLN was chosen as the terminal preparation for further therapeutic studies.

These results confirmed that BBB crossing ability does not linearly correlate with ligand density. Low ligand density would lead to insufficient affinity to receptors, while too high ligand density would cause steric hindrances by closely adjacent ligands and increase nonspecific interactions with peripheral organs, resulting in reduced intracerebral accumulation. The optimized ligand density could maximize BBB penetration and subsequent effectively accumulated in lesion location. Once get rid of BBB limitation, APLN/MB established the foundation that APLN delivered MB into neurons for inhibiting Tau phosphorylation and the carrier itself executing A*β* clearance, and then achieved “Drug‐Carrier” synergy therapy. In addition to Ang peptide, apolipoprotein E peptide [ApoE, (LRKLRKRLL)2C] can specifically bind to low‐density lipoprotein receptor members (LDLRs), including LDL receptor (LDLR) and LDLR‐related proteins 1 and 2 (LRP1 and LRP2) for BBB crossing.^[^ ^32^ ^]^ Similarly, the optimized ligand density of ApoE can maximize BBB penetration of nanoparticles. Moreover, considering the LRP1 reduction in some pathological state,^[^ ^33^ ^]^ codelivery drugs that can raise LRP1 expression is another strategy to improve the BBB penetration of LRP1‐targeted nanoparticles.^[^ ^34^ ^]^

### 2.5 Carrier‐Mediated A*β* Clearance through Microglia Uptake and Degradation

**Figure 4:**
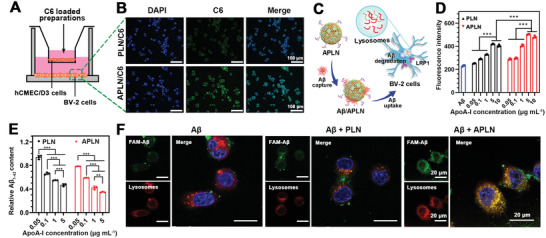
Microglial targeting and the promotion of microglial uptake and degradation of A*β*. A) Schematic illustration of BBB Transwell model. B) Fluorescence images of cellular uptake of PLN/C6 and APLN/C6 in microglia, respectively. Scale bar = 100 µm. C) Scheme of APLN promoting A*β* uptake and degradation by microglia. D) Cellular uptake of FAM‐A*β*_1‐42_ in microglia in the absence or presence of PLN and APLN at different apoA‐I concentrations, respectively (*n* = 3). E) Relative intracellular A*β* were quantified by ELISA after coincubation with A*β* and APLN or PLN (*n* = 3). F) CLSM images showing FAM‐A*β*_1‐42_ clearance by lysosomal network in the absence or presence of APLN (or PLN) after incubation for 4 h, respectively. Scale bar = 20 µm. Data were presented as mean ± SD. **p < 0.01 and ***p < 0.001.

The endocranial resident microglia plays a crucial role in central A*β* clearance. However, when A*β* contact with microglia, it can destroy the membrane with cell damage, leading to sacrificed A*β* clearance efficacy.^[^ ^35^ ^]^ The high A*β* binding of APLN/MB promoted A*β* capture and form nonpathogenic complex, which could alleviate A*β*‐cell adhesion with reduced membrane damage towards both neuron and microglia (Figure S4, Supporting Information). Moreover, microglia could recognize and uptake APLN/MB through scavenger receptor class B type I (SR‐BI) by apoA‐I and Ang of nanocomposites for enhanced A*β* clearance. To verify this process, we tested whether APLN/MB can cross BBB, followed by microglia internalization. As demonstrated in **Figure** **4**A, a Transwell BBB model was established by coincubation of cerebral microvascular endothelial cell (hCMEC/D3) on the upper chamber and microglia (BV‐2 cells) on the lower chamber, respectively. Transmembrane resistance (TEER) of the cell monolayer was measured every day to indicate the formation of hCMEC/D3 monolayers. When TEER value reached 50 Ω cm^−2^ and remained stable (Figure S5, Supporting Information),^[^ ^31^ ^]^ various C6‐loading preparations were added in the upper chamber. To evaluate BBB permeability and cellular internalization, fluorescence images of BV‐2 cells on the lower chamber were visualized under inverted fluorescence microscope. As shown in Figure 4B, BV‐2 cells treated with APLN/C6 exhibited significantly higher fluorescence intensity than those treated with PLN/C6, indicating that BBB permeability and microglia endocytosis of APLN/C6 could be improved with the assistance of *α*Ang. We further investigated the ratio of APLN/MB uptake by microglia and neuronal cells. As shown in Figure S6 of the Supporting Information, APLN/MB had about 2.1‐fold more uptake by BV‐2 cells than SH‐SY5Y cells. In further consideration of that the number of neurons in mice brain is 55‐fold more than microglia,^[^ ^36^ ^]^ APLN/MB might provide ≈26.2 times more uptake by microglia than neurons in vivo. Interestingly, after A*β* oligomer capture, the uptake of APLN/C6/A*β* by microglia was increased 3.2‐fold to neuron cells, suggesting that A*β*‐mediated more APLN/C6 uptake by microglia for effective clearance. The enhanced microglia uptake of APLN/C6/A*β* might be ascribed to the recognition of A*β* by the microglial membrane receptors for A*β*‐binding, including the scavenger receptors, the receptor for advanced glycation end product, and so on.^[^ ^37^ ^]^

According to the confirmed BBB traverse and microglia targeting capability of the preparations, we further evaluated the effect of PLN and APLN carrier on promoting A*β* uptake and degradation by microglia (Figure 4C). BV‐2 cells were coincubated with FAM‐A*β*_1‐42_ oligomer in the presence of APLN (or PLN) to determine the cellular uptake of A*β*. As illustrated in Figure 4D, the cellular uptake of A*β* increased with apoA‐I concentration raised from 0.05 to 5 µg mL^−1^, indicating that PLN could improve A*β* uptake in an apoA‐I concentration‐dependent manner. However, the efficient internalization of A*β* was not uplifted when the concentration further increased to 10 µg mL^−1^. These results suggested that PLN could promote A*β* endocytosis through SR‐BI receptor and approach saturation at the concentration of 5 µg mL^−1^, which was probably limited by maximal amount of microglia uptake. Moreover, cells treated with APLN gave similar increase tendency and reached the maximum at apoA‐I concentration of 5 µg mL^−1^, which possessed a higher amount of A*β* uptake compared with those treated PLN at the same apoA‐I concentration. This result suggested that APLN could extrarecognize LRP1 receptor (*α*Ang binding) in microglia that was independent of SR‐BI receptor (apoA‐I binding) to enhance A*β* uptake. To further analysis the microglial degradation of A*β* in the presence of APLN (or PLN), the intracellular A*β* was detected by ELISA and presented as relative A*β* content after another incubation for 4 h. As shown in Figure 4E, even with higher amount of A*β*_1‐42_ internalized into microglia with the help of APLN, about 1.65‐fold of A*β* degradation was observed when compared with that of A*β* only control group.

Lysosome was the primary organelle of microglia utilized for A*β* clearance and degradation. To further investigate the mechanism of ALPN carrier in promoting A*β* degradation, the colocalization of A*β*_1‐42_ with lysosome was measured through confocal laser scanning confocal microscope (CLSM). As shown in Figure 4F, cells in APLN group presented intense yellow fluorescence, indicating the colocalization of green FAM‐A*β*_1‐42_ fluorescence and red lysosome fluorescence. The results further proved that APLN could facilitate A*β* clearance through the lysosomal network of microglia, explaining the enhanced A*β* degradation. The similar results were observed in the presence of APLN/MB or PLN/MB (Figures S7–S9, Supporting Information), demonstrating that APLN/MB or PLN/MB held the A*β* capability of carrier (APLN or PLN) after MB loading. The collective evidence confirmed that APLN/MB could efficiently capture and accelerate microglia‐mediated A*β* uptake and degradation in the lysosome. The mission of A*β* clearance was executed primarily by carrier of APLN/MB, which was crucial in achieving “Drug‐Carrier” synergy therapy for AD.

### 2.6 Neuroprotection after Inhibition of Tau Hyperphosphorylation and Aggregation

**Figure 5:**
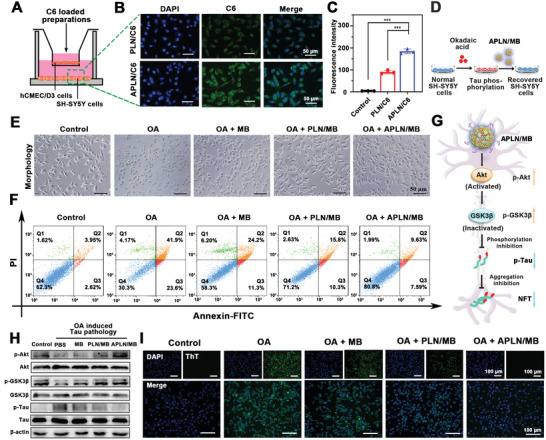
Neuron targeting and Tau phosphorylation inhibition via Akt/GSK3*β* pathway. A) Schematic illustration of in vitro BBB model. B) Cellular uptake of PLN/C6 and APLN/C6 in SH‐SY5Y cells after crossing BBB, respectively. Scale bar = 50 µm. C) Flow cytometric analysis of PLN/C6 and APLN/C6 uptake on SH‐SY5Y cells (*n* = 3). D) Schematic illustration of establishing tauopathy cell model by OA, followed by the treatment of APLN/MB. E) Morphological photographs of OA (40 nmol L^−1^) induced tauopathy SH‐SY5Y cells after treated with MB, PLN/MB, and APLN/MB, respectively. Scale bar = 50 µm. F) Apoptosis analysis on OA (40 nmol L^−1^) induced tauopathy SH‐SY5Y cells after treated with MB, PLN/MB, and APLN/MB as measured by flow cytometry, respectively. G) Scheme of mechanisms of the inhibition of Tau phosphorylation by Akt/GSK3*β* pathway. H) Western blot for p‐Akt, Akt, p‐GSK3*β*, GSK3*β*, p‐Tau, and Tau in SH‐SY5Y cells and OA prestimulated SH‐SY5Y cells, followed by the treatment of different preparations. I) Fluorescence images of Tau protein aggregation using ThT probe. Scale bar = 100 µm. Data were presented as mean ± SD. ***p < 0.001.

Tested in clinical studies, MB could downregulate p‐Tau level and inhibit Tau protein aggregation.^[^ ^38^ ^]^ However, the further application is limited by the low efficiency of BBB permeation and serious side effects that result from a lack of selectivity for diseased cells. To confirm that APLN/MB could target and accumulate in neuronal cells after crossing BBB and then inhibit Tau‐associated AD pathogenesis, SH‐SY5Y cells were cultured in the lower chambers of the Transwell model as described above (**Figure** **5**A). As shown in Figure 5B, cells treated with APLN/C6 gave more intense green fluorescence (C6) that distributed evenly in the cytoplasm than PLN/C6 group. The quantitative fluorescence intensity of APLN/C6 group was 1.89‐fold higher than that of PLN/C6 group (Figure 5C), suggesting that APLN/C6 could effectively traverse through BBB with integral structure and was further endocytosed by SH‐SY5Y cells. In summary, Ang modified nanocomposites could mediate cargo cross BBB and accumulate in neurons, which might suppress Tau hyperphosphorylation and prevent neuronal apoptosis.

To establish the tauopathy cell model, various concentrations of OA were applied for SH‐SY5Y cell treatment to induce Tau pathology, and cell viability was detected by MTT assay (Figure 5D).^[^ ^39^ ^]^ As shown in Figure S10 of the Supporting Information, the most acute neuron cell damage with ≈40% of cell viability was observed in 40 nmol L^−1^ of OA treated SH‐SY5Y cells, indicating the optimal concentration of OA to induce Tau pathology, which was used to induce tauopathy cell model for exploring the neuroprotection mechanisms of APLN/MB. As displayed in Figure 5E, OA could induce toxicity to SH‐SY5Y cells, resulting in abnormal morphology of SH‐SY5Y cells and impaired proliferation of cells. After treatment with APLN/MB, the shrinkage and swelling of cells were ameliorated. Once Tau hyperphosphorylation and aggregation were inhibited, the Tau pathology of neuron cells could be rescued for neuron protection. To further investigate the neuroprotective effect of APLN/MB in the tauopathy model cells, the cell viability was also detected by MTT assay (Figure S11, Supporting Information). The cell viability treated with free MB increased from 40.31% to 57.79% when MB concentration was higher than 1 µg mL^−1^; while cells treated with PLN/MB displayed higher viability of 65.87%. As expected, APLN/MB treatment increased cell viability to 71.03% because that LRP1‐mediated recognition of Ang on APLN/MB could facilitate more cellular internalization of MB. These results demonstrated that APLN/MB could improve the protective effect of MB through targeting and accumulation in tauopathy model cells. Moreover, the analysis of SH‐SY5Y cell apoptosis after Annexin V‐FITC/PI staining confirmed the capability of APLN/MB to prevent OA‐induced apoptosis. As shown in Figure 5F, APLN/MB treatment significantly decreased the cell apoptosis rate to 17.22%, which was lower than free MB treatment (≈35% of apoptosis) and PLN/MB treatment (≈27% of apoptosis), respectively. The results demonstrated that APLN/MB could effectively protect neurons from OA‐induced apoptosis of Tau pathology.

### 2.7 Akt/GSK3*β* Signaling Pathway Mediating Inhibition of Tau Hyperphosphorylation and Aggregation

Akt/GSK3*β* signaling pathway is considered as an important role in Tau pathogenesis.^[^ ^40^ ^]^ To further investigate the underlying mechanism of APLN/MB on inhibiting Tau pathology, western blot was performed to evaluate Tau associated proteins and signal pathways. As shown in Figure 5G, a Tau kinase of glycogen synthase kinase3*β* (GSK3*β*) promotes Tau phosphorylation into p‐Tau in AD pathology. However, in the normal physiological condition, GSK3*β* could be inactivated with residue Ser9 residue phosphorylated into p‐GSK3*β* (inactive state) by the phosphorylated protein kinase B (p‐Akt), which was an upstream kinase of GSK3*β*. As expected, OA treatment could effectively downregulate the expression of both p‐Akt and p‐GSK3*β*, leading to the over‐phosphorylation of Tau into p‐Tau, indicating the formation of Tau pathology cell model (Figure 5H). However, the activation of Akt/GSK3*β* signaling pathway with increased p‐Akt and p‐GSK3*β* expression could be observed after MB treatment, demonstrating that MB could attenuate Tau pathology through Akt/GSK3*β* signaling pathway.^[^ ^41^ ^]^ When compared with PLN/MB, APLN/MB treatment could significantly increase the expression of p‐Akt and p‐GSK3*β* with reduced p‐Tau level that was the similar as that of normal cells (Figure S12, Supporting Information). The results indicated that *α*Ang could specifically recognize LRP1 receptors on SH‐SY5Y cells to improve the intracellular distribution of MB. Taken together, APLN/MB could remarkably promote the activation of the Akt/GSK3*β* pathway because that APLN carrier could enhance the drug accumulation in damaged cells, which eventually reduced the hyperphosphorylated Tau protein for neuron protection.

Phosphorylated Tau proteins can aggregate intracellularly to form neurofibrillary tangles, leading to neuron damage. To investigate the capability of APLN/MB in inhibiting Tau aggregation, thioflavin T (ThT) was employed to detect the aggregate form of Tau. As a *β*‐sheet tracer agent, ThT could bind with *β*‐sheet of Tau aggregation with sharply increased fluorescence intensity. Hence, the intensity of green fluorescence (ThT) indicated the degree of Tau aggregation. As shown in Figure 5I, when cells were prestimulated with OA, a significantly increased fluorescence intensity of ThT could be observed, indicating that OA could effectively induce Tau aggregation. However, the fluorescence intensity became weak in the presence of MB, suggesting that Tau aggregation was restrained due to the inhibition of Tau phosphorylation. By contrast, an obvious decrease in fluorescence intensity was observed after treatment with APLN/MB, demonstrating that APLN/MB possessed a high efficiency in the inhibition of Tau aggregation, consistent with the results of p‐Tau level regulated by the Akt/GSK3*β* pathway. These results confirmed that MB‐loaded nanoparticles could inhibit Tau aggregation based on the activation of Akt/GSK3*β* pathway, and prevent the formation of NFTs. As designed, APLN/MB has completed the other vital procedure of “Drug‐Carrier” synergy therapy, namely, drug of MB could accumulate efficiently in tauopathy cells with the help of APLN carrier, and eventually inhibit Tau phosphorylation and p‐Tau aggregation for blocking progression of Tau pathology.

### 2.8 Amelioration of Learning and Memory Impairments in Dual‐Pathology AD Models

**Figure 6:**
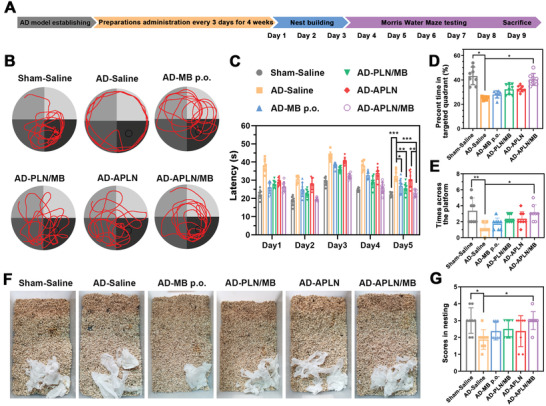
Different formulations improved cognitive and memory impairments of AD model mice. A) Time scheme of AD model establishment, drug therapy and therapeutic assessment. B) The representative path tracings of mice in the MWM test. C) Latency (*n* = 8). D) Percent time spend on the targeted quadrant (*n* = 8). E) Times across the platform (*n* = 8). F) Representative images of nest behavior. G) Scores for each group (*n* = 8). Data were presented as mean ± SD. *p < 0.05, **p < 0.01, and ***p < 0.001.

To determine whether APLN/MB could ameliorate cognitive and memory impairments, the established A*β*–Tau copathological AD model mice were intravenously administrated with MB preparations at drug equivalent dose of 2 mg kg^−1^, APLN at the equivalent lipid concentration, and intragastrically administered free MB solution at 20 mg kg^−1^ every 3 days for 4 weeks, respectively (**Figure** **6**A). The cognitive and memory improvement of mice were examined through MWM test and nesting behavior, respectively.^[^ ^42^ ^]^ In MWM experiment, AD mice treated with saline showed poorly platform searching performance as expected and obvious deficits in spatial learning with the longest escape latency time (Figure 6B,C), indicating that the coinjection of A*β*_1‐42_ and OA in bilateral hippocampus could effectively trigger learning and memory deficit. By contrast, AD mice treated with MB p.o. showed decreased escape latency time of 27 s, while those treated with APLN/MB displayed a significant reduction in term of latency time (≈21 s). This result indicated that free MB could mildly improve memory and cognition, while APLN/MB achieved a stronger therapeutic effect due to the high BBB permeation of APLN/MB via Ang targeting ligand. After treatment with PLN/MB, AD mice presented the increased percentage of time spent in the target quadrant and more numbers of platform crossings (Figure 6D,E), which were slightly higher than MB treated mice. By contrast, AD mice administered with APLN displayed more time spent in the targeted platform quadrant than that of AD mice treated with saline, demonstrating that drug‐free APLN could execute the “Carrier” mission to cross BBB for A*β* clearance and improve learning performance of AD mice. Compared with APLN administration, AD mice treated with APLN/MB performed obvious enhancement in searching platform with a higher percentage of time in the target quadrant (40%) and more numbers of across platform (3.5 times). The results indicated that APLN/MB could sequentially execute “Carrier” mission for crossing BBB to remove A*β*, and implement “Drug” function for ameliorating Tau related pathology, eventually achieving “Drug‐Carrier” synergy therapy to improve the cognitive performance of AD mice. Afterward, the nesting behavior was performed to assess the hippocampal function of AD mice after treatment. As shown in Figure 6F, AD mice failed to build a complete and mass nest because of cognitive deficiency. However, AD mice treated with APLN/MB displayed excellent nesting skills that the nest was concentrated on one side and gathered. Moreover, AD mice treated with APLN/MB gained a similar score to that of the Sham mice (Figure 6G), which was higher than that of other groups. These results confirmed that APLN/MB nanoparticles could remarkably recover spatial memory disorder and improve the cognitive performance of AD model mice.

### 2.9 Reduction of A*β* Deposition and Inhibition of Tau Hyperphosphorylation in AD Mice

**Figure 7:**
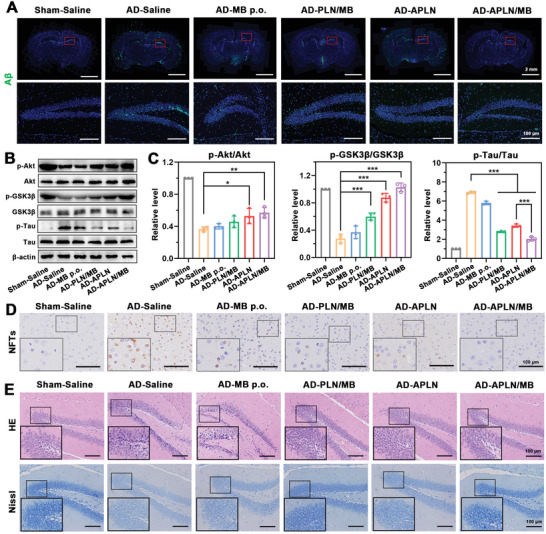
Evaluation of A*β* level, p‐Tau level, and neuronal morphology. A) Immunostaining of A*β* in the whole brain and representative images of hippocampal DG region of Sham mice and AD model mice administered with saline and different formulations for 4 weeks. Green represents A*β*_1‐42_ stained by antibody 6E10, blue represents nuclei stained by DAPI. Scale bars = 2 mm or 100 µm. B) Western blot results of p‐Akt, Akt, p‐GSK3*β*, GSK3*β*, p‐Tau, and Tau in mice brains. C) Quantification results of p‐Akt, p‐GSK3*β*, and p‐Tau. p‐Akt, p‐GSK3*β*, and p‐Tau were normalized to total Akt, GSK3*β*, and Tau, respectively (*n* = 3). D) Representative images of NFTs in the cortex region of Sham mice and AD model mice administered with saline and different formulations for 4 weeks. Scale bars = 100 µm. E) Representative images of HE and Nissl staining in the DG region of Sham mice and AD model mice administered with saline and different formulations for 4 weeks. Scale bars = 100 µm. Data were presented as mean ± SD. *p < 0.05, **p < 0.01, and ***p < 0.001.

Encouraged by the results that APLN/MB could promote microglial phagocytosis of A*β* and inhibit Tau phosphorylation in vitro, immunofluorescence and western blot were employed to assess the effects on eliminating A*β* deposition and inhibiting phosphorylated Tau in AD model mice, respectively. As illustrated in **Figure** **7**A, there was a massive A*β* burden in the brain of AD mice, suggesting that bilateral intrahippocampal coinjection of A*β*_1‐42_ and OA could induce effective A*β* accumulation. After MB p.o. treatment, A*β* deposits mildly reduced because that MB could only slightly reduce A*β* deposits by regulating Tau pathology. By contrast, APLN treatment obviously decreased A*β* deposits than that of AD‐saline mice, suggesting that APLN could remove A*β* in AD mice due to the carrier function with A*β* binding affinity. In sharp contrast, A*β* burdens in the DG region of AD mice were significantly reduced and even almost disappeared after APLN/MB treatment. Similar efficacy in the CA1, CA3, and cortex regions were also observed after APLN/MB treatment for four weeks (Figure S13, Supporting Information), confirmed that APLN/MB could promote A*β* clearance ability.

To further evaluate synergy therapy mechanisms, the expression of phosphorylated Tau (p‐Tau) and associated protein of Akt/GSK3*β* pathway were tested by western blot. As shown in Figure 7B,C, compared with the Sham mice, the level of p‐Akt and p‐GSK3*β* in AD mice treated with saline was significantly reduced. By contrast, the Akt/GSK3*β* pathway was inactived with increased p‐Tau level in AD mice. Due to the restriction of BBB, free MB performed limited effects on the activation of Akt/GSK3*β* and downregulation of p‐Tau level. Nevertheless, when compared with AD mice treated with saline, significantly decreased p‐Tau expression was observed in APLN‐treated AD mice. The results indicated that A*β* clearance mediated by APLN (carrier) could effectively inhibit Tau phosphorylation. Moreover, compared with APLN group, APLN/MB treatment could more effectively active Akt/GSK3*β* pathway with reduced Tau phosphorylation, which was attributed to the synergy of MB (drug) and A*β* clearance (carrier). To investigate whether p‐Tau inhibition could hinder the formation of neurofibrillary tangles in the AD mice brains, the immunohistochemical analysis of brains slices were further evaluated. Compared with the sham mice, an obvious p‐Tau aggregation of neurofibrillary tangles was observed in AD mice brain (Figure 7D; Figure S14, Supporting Information). The treatment with APLN could slightly decrease p‐Tau aggregation due to that A*β* clearance might play positive roles on the inhibition of Tau phosphorylation. By contrast, no obvious p‐Tau aggregation could be found in AD mice treated with APLN/MB. The encouraging results might be ascribed to the combination of Tau phosphorylation inhibition by drug (MB) and A*β* clearance by carrier (APLN). The results indicated that APLN/MB could eliminate A*β* and inhibit Tau phosphorylation according to “Drug‐Carrier” synergy therapy.

### 2.10 Ameliorating and Rescuing Damaged Neurons in AD Mice

The toxic A*β* and phosphorylated Tau work together to induce neurotoxicity. To evaluate histological features of mice treated with various preparations, HE and Nissl staining were performed to stain nerve cells in the brains. As depicted in Figure 7E and Figure S15 (Supporting Information), markable neuronal shrinkage, cytoplasmic condensation, and nucleus pyknosis were observed in the CA1, CA3, and DG regions of hippocampus and cortex of AD mice. By contrast, APLN/MB treatment obtained neuron protection and significantly alleviated neuron damage, while other groups gave no obvious therapeutic effect. Nissl bodies were collections of granular endoplasmic reticulum and ribosomes in nerve cells, which could be stained with strong‐color in normal nerve cells and essential for the evaluation of neuroprotection.^[^ ^43^ ^]^ Compared with Sham group, neuronal hypocellularity and lighter‐color were observed in the hippocampus and cortex of AD mice treated with saline (Figures S16 and S17, Supporting Information), indicating the decrease of neuron viability in AD mice. By contrast, APLN/MB treatment significantly ameliorated the neuronal damage, while PLN/MB treatment did not provide such obvious amelioration, demonstrating that BBB permeation of drug and carrier is indispensable for the rescue of neuronal integrity and neuron loss. All the above results demonstrated that APLN/MB performed site‐specific shuttling and superior therapeutic efficacy in vivo. Moreover, HE staining was evaluated for the safety profiles of MB‐loaded nanoparticles. These results confirmed no pathological changes in the major organs (Figure S18, Supporting Information), which were consistent with the evaluation of cell safety (Figures S19–S21, Supporting Information). The results indicated the desirable safety of the biomimetic lipid nanocomposites for the synergy therapy of AD.

## 3 Conclusion

In summary, a biomimetic protein–lipid nanocomposite of APLN/MB was successfully assembled with endogenous apoA‐I and lipids together with MB shielding to achieve “Drug‐Carrier” synergy in AD therapy. Inspired by the function and structure of HDL, APLN carrier was designed to mediate MB for inhibiting tau phosphorylation and itself performing antibody‐like high A*β* binding affinity for accelerating A*β* clearance. In A*β*–Tau copathological AD model mice, the high BBB penetration was achieved by optimizing Ang ligand density for APLN/MB surface modification. Compared with single pathogenesis targeted therapy, “Drug‐Carrier” synergy therapy strategy could eliminate A*β* and inhibit tau protein phosphorylation simultaneously. Moreover, the therapeutic effects were more remarkable displayed in reversing the memory and cognitive decline in AD mice. As a result, the biomimetic lipid nanocomposite contributed to exploring the possibility to enhance BBB cross and open up a new avenue for AD treatment.

## 4 Experimental Section

The experimental section is available in the Supporting Information.

## Conflict of Interest

The authors declare no conflict of interest.

## Supporting information

Supporting Information

## Data Availability

The data that support the findings of this study are available from the corresponding author upon reasonable request.
