# Supplementary material for: “Drug‐Carrier” Synergy Therapy for Amyloid‐β Clearance and Inhibition of Tau Phosphorylation via Biomimetic Lipid Nanocomposite Assembly
Source: Adv Sci (Weinh). 2022 Mar 20;9(14):2106072. doi: 10.1002/advs.202106072 (PMC9108666; doi:10.1002/advs.202106072)
Supplement: Supplementary file 1 — Supporting Information [file ADVS-9-2106072-s001.pdf]

ADVANCED SCIENCE
Open Access

Supporting Information

for *Adv. Sci.*, DOI 10.1002/advs.202106072

“Drug-Carrier” Synergy Therapy for Amyloid-β Clearance and Inhibition of Tau Phosphorylation via Biomimetic Lipid Nanocomposite Assembly

Guochen Han, Kaiwen Bai, Xiaoyu Yang, Chenhua Sun, Yi Ji, Jianping Zhou, Huaqing Zhang* and Yang Ding**

Supporting Information

"Drug-Carrier" Synergy Therapy for Amyloid-β Clearance and Inhibition of Tau Phosphorylation via Biomimetic Lipid Nanocomposite Assembly

Guochen Han, Kaiwen Bai, Xiaoyu Yang, Chenhua Sun, Yi Ji, Jianping Zhou, Huaqing Zhang*, Yang Ding**

Contents

Table S1. Characterization of the PLN/MB and APLN/MB.

Table S2. The average binding affinity to Aβ monomer and oligomer.

Figure S1. The binding affinity of 4F peptide and 4F-Angiopep-2 (αAng) to lipid nanoparticles.

Figure S2. Representative images and scores of nest behavior for each group.

Figure S3. Quantification results of p-Akt, p-GSK3β and p-Tau in mice brains.

Figure S4. Cell viability of both BV-2 cells and SH-SY5Y cells in the presence of Aβ and APLN/MB/Aβ.

Figure S5. The growth curve of the hCMEC/D3 cell monolayer TEER value.

Figure S6. The ratio of APLN/MB uptake by BV-2 cells and SH-SY5Y cells.

Figure S7. Cellular uptake of FAM-A$\beta_{1\text{-}42}$ in microglia in the absence or presence of PLN/MB and APLN/MB.

Figure S8. Intracellular relative Aβ were quantified by ELISA.

Figure S9. CLSM images for colocalization analysis of FAM-A$\beta_{1\text{-}42}$ with lysosome in microglia in the presence of PLN/MB and APLB/MB.

Figure S10. Cell viability of SH-SY5Y cells after co-incubated with okadaic acid.

Figure S11. Protection effect of MB, PLN/MB and APLN/MB on OA induced cytotoxicity of SH-SY5Y cells.

Figure S12. Quantification results of p-Akt, p-GSK3β and p-Tau in SH-SY5Y cells after treatment with MB, PLN/MB and APLN/MB.

Figure S13. Immunostaining of Aβ deposition in the CA1, CA3 and cortex region of Sham mice or AD model mice treated with saline or different preparations.

Figure S14. Representative images of NFTs in the DG region of Sham mice and AD model mice administered with saline and different formulations.

Figure S15. HE staining of neurons in the CA1, CA3 and cortex region of Sham mice or AD model mice treated with saline or different preparations.

Figure S16. Nissl staining of neurons in the CA1, CA3 and cortex region of Sham mice or AD model mice treated with saline or different preparations.

Figure S17. Enlarged Nissl staining imaging of neurons in the DG region.

Figure S18. HE staining of the major organs of Sham mice or AD model mice treated with saline or different preparations.

Figure S19. The cell viability of hCMEC/D3 cells treated with different concentrations of different preparations.

Figure S20. The cell viability of BV-2 cells treated with different concentrations of different preparations.

Figure S21. The cell viability of SH-SY5Y cells treated with different concentrations of different preparations.

Experimental Section

Materials

MB was obtained from Shanghai Macklin Biochemical Co., Ltd. (Shanghai, China). αAng peptide (AC-FAEKFKEAVKDYFAKFWD-GSG-TFFYGGSRGKRNNFKTEEY) was purchased from Guoping Pharmaceutical Co., Ltd. (Anhui, China). Amyloid beta 1-42 ($A\beta_{1-42}$), coumarin-6 (C6) and DiR were bought from Sigma-Aldrich Co., Ltd. (St. Louis, MO, USA). Okadaic acid (OA) was obtained from Cayman Co., Ltd. (Jiangsu, China). Lyso Tracker-Red was bought from Invitrogen Corporation (Carlsbad, CA, USA). Hoechst33258 was obtained from Kaiji Biotechnology Co., Ltd. (Jiangsu, China). Dulbecco's modified eagle's medium (DMEM) and fetal bovine serum (FBS) were from Gibco (Grand Island, NY, USA). All other reagents were used in analytical grade.

Cells

The brain endothelial hCMEC/D3 cells, microglia BV-2 cells and SH-SY5Y neuroblastoma cells were from the Cell Bank of the Chinese Academy of Sciences (Shanghai, China). Cells were cultured in DMEM with 10% FBS at 37 °C in a humidified atmosphere with 5% CO_2.

Animals and AD mice model

ICR mice (18-22 g) were purchased from Qinglongshan Animal Center (Jiangsu, China). The mice were housed and maintained in a 12-hour light/dark regular environment and complied with the National Institute of Health Guide for the Care and Use of Laboratory Animals and China Pharmaceutical University Ethics Committee.

Recent advances have demonstrated that the triple-transgenic (3xTg) models can express three AD-related transgenes, including amyloid beta precursor protein (APPswe), presenilin 1 ($PS1^{M146V}$), and Tau (Tau^{P301L}) with the accumulation of neurotoxic Aβ plaques and Tau NFTs in the brain.[1] However, the 3xTg model costs enormous money and time because both Aβ and Tau pathology cannot be present in the brain until 9 months of age,[2] which limits the application in studies. According to previous reports, $A\beta_{1-42}$ (82 μmol L^{-1}) was incubated in saline at 37 °C for 7 days to aggregate into $A\beta_{1-42}$ fibers.[3] To find out a feasible modeling dose of AD mice with two pathological markers ($A\beta_{1-42}$ aggregation and phosphorylated Tau), we referred to the $A\beta_{1-42}$ dose we reported before and set a series of OA concentrations of 1 μmol L^{-1}, 2 μmol L^{-1} and 4 μmol L^{-1},[4] which named as Low, Middle and High modeling group, then mixed three concentrations of OA with $A\beta_{1-42}$, respectively. According to the aggregation of $A\beta_{1-42}$ and phosphorylated Tau, the best modeling group was selected and used in subsequent experiments.

$A\beta_{1-42}$ (5 μL, 82 μmol L^{-1}) with 1 μL of OA (three different concentrations of 1 μmol L^{-1}, 2 μmol L^{-1} and 4 μmol L^{-1}) were mixed together, respectively, then slowly injected into the bilateral hippocampus (anteroposterior -2.5 mm, mediolateral ±1.5 mm and dorsoventral -2.0 mm). Saline-only injected mice were the sham control. Before the treatment and in vivo imaging, all the mice were given a week to recover.

Preparation of APLN/MB

For PLN/MB preparation, 30 mg of phosphatidylcholine (PC) was dissolved in 2 mL of mixed solvent ($CH_3OH/CHCl_3$ = 1/1, v/v) and then removed solvent to form a lipid film by rotary evaporation. At the same time, 1.2 mg of methylene blue (MB) was dissolved in 1 mL of water for hydrating. After probe sonicated (Beidi-900TE, Nanjing Beidi Experimental Instrument Co., Ltd., Jiangsu, China) at 200 W for 10 min in an ice water bath, the solution was co-incubated with apoA-I (3 mg) at 37 °C for 12 h. APLN/MB was prepared as follows: αAng (1.5 mg) was added to the PLN/MB emulsion, stirred for another 12 h. The C6-loaded and DiR-loaded nanocomposites were prepared in the same way by replacing MB with C6 and DiR for tracking in vitro and in vivo, respectively

Characterization

The morphology of nanocomposites was visualized by transmission electron microscopy (TEM, H-600, Hitachi, Ltd., Tokyo, Japan). For TEM analysis, preparations were added onto a copper grid, followed by phosphotungstic acid negative staining and drying with an infrared lamp. The particle size, polydispersity index (PDI) and zeta potential of nanocomposites were

measured by dynamic light scattering (DLS, Zetasizer, Malvern Panalytical Ltd., Malvern, UK), respectively. To analyze the entrapment efficiency (EE%) and the drug loading (DL%), MB-loaded nanocomposites were dissolved in methanol and ultrasound (20 min) to release MB, after free MB was removed by centrifugation at 8000 rpm for 10 min. The absorbance of MB was determined by UV-spectrophotometer (TU-1810, Beijing Universal Instrument Co., Ltd., Beijing, China) at 653 nm.

The EE% and DL% were calculated as follow: EE% = (the quality of MB in nanocomposites)/(the quality of MB fed initially) × 100%; DL (%) = (the quality of MB in nanocomposites)/(the quality of drug-loaded nanocomposites) × 100%

In vitro release analysis

In vitro release analysis was conducted with phosphate buffered saline (PBS, pH 7.4) solution as a release medium. The 2 mL of MB, PLN/MB and APLN/MB were suspended in a dialysis membrane bag (MWCO = 3500 Da), sunken and released in 50 mL of PBS solution at 100 rpm agitation for 48 h, respectively. The release medium (2 mL) was collected at the predetermined interval, while a fresh medium was supplied at the same volume. The MB in the collected medium was analyzed by UV-spectrophotometer. The cumulative release (CR%) of MB loaded in nanocomposites was calculated as a ratio to the total MB with the following equations: CR% = (cumulative amount of MB in the release medium)/(total amount of MB in nanocomposites) × 100%

Stability of PLN/MB and APLN/MB

To evaluate the stability of PLN/MB and APLN/MB in physiological conditions, the PLN/MB and APLN/MB were suspended in PBS (pH 7.4) containing 10% FBS, respectively. During incubation at room temperature for 96 h, the changes of particle size andPDI were measured by DLS.

Surface plasmon resonance (SPR) analysis of Aβ binding affinity

The $A\beta_{1-42}$ peptide was dissolved in hexafluoroisopropanol (HFIP), evaporated by nitrogen to form the $A\beta_{1-42}$ film. To prepare the $A\beta_{1-42}$ monomer, dimethyl sulfoxide (DMSO) was added, and then 5 mmol L^{-1} of $A\beta_{1-42}$ was dissolved ultrasonically in an ice bath for 10 min. $A\beta_{1-42}$ oligomer was prepared using monomeric $A\beta_{1-42}$ (100 μmol L^{-1}) incubated in the 37°C PBS atmosphere for 24 h. The FAM-$A\beta_{1-42}$ was prepared by using the same way.

The Open SPR instrument (Nicoya, Ltd., ON, Canada) was used to conduct surface plasmon resonance (SPR) analysis of the binding ability between nanocomposites and two forms of $A\beta_{1-42}$ aggregation.

The sensor chip was activated by injecting N-hydroxysuccinimide (NHS) and Ethyl (dimethylaminopropyl)-carbodiimide (EDC, 1/1, mol/mol), then 200 μL of $A\beta_{1-42}$ solution was introduced to reach the appropriate level of immobilization. The carboxyl group on the chip was blocked by injecting ethanolamine (pH 8.5). When the SPR instrument started, the flow rate of the running buffer which is PBS (0.01 mol L^{-1}, pH 7.0) was set at 20 μL min^{-1}. Meanwhile, the regeneration buffer sodium dodecyl sulfate (SDS) solution (0.01 mol L^{-1}) was running at 150 μL min^{-1}. Before the next injection, the sensor chip was equilibrated by PBS for 5 min. The binding ability between series concentrations of the nanocomposites and $A\beta_{1-42}$ were assessed and analyzed through Trace Drawer software.

SPR analysis of dynamic binding behavior of peptide and lipid vesicles

The LIP-1 sensor chip was equilibrated at room temperature, dock it into the apparatus and rinsed with PBS (0.01 mol L^{-1}, pH 7.0). Set the flow rate to 150 μL min^{-1} and precondition the surface with injections of 80% isopropanol. 200 μL of 0.5 mg mL^{-1} lipid vesicles was injected and immobilized at the slow flow rate (20 μL min^{-1}). Thereafter, 4F peptide, Ang peptide and αAng were injected at series concentrations for recording binding behavior. There results were assessed and analyzed through Trace Drawer software.

Cytotoxin of PLN/MB and APLN/MB

To evaluate the cytotoxin of PLN/MB and APLN/MB towards cells, the hCMEC/D3 cells, the SH-SY5Y cells and BV-2 cells were seeded onto 96-well plates and cultured for 12 h, respectively. Then, the cultured medium was replaced by an FBS-free medium with PLN/MB and APLN/MB at a series of lipids concentrations of 0.1, 1, 10, 25, 50, 100 and 200 μg mL^{-1} for another 48 h, respectively. The cells incubated with FBS-free medium were usded as control. Subsequently, 100 μL of 0.5% 3-(4,5-Dimethyl-2-Thiazolyl)-2,5-Diphenyl tetrazolium bromide (MTT) solution was added in the plates. After incubated for 4 h, DMSO was added to replace the medium and incubated for 10 min. The absorbance was measured by a microplate reader (Varioskan LUX, Thermo Fisher Scientific, Inc., MA, USA) at 490 nm. The following equation calculated the viability of cells: cell viability (%) = O.D.sample /O.D.control × 100%

Cellular internalization assay

To track nanocomposites in the hCMEC/D3 cells, MB was replaced by coumarin-6 (C6) to obtain PLN/C6 and APLN/C6 with a series αAng concentrations of 1%, 5%, 10% and 15% of lipid quality, respectively. The hCMEC/D3 cells (1×10^5 cells well^{-1}) were cultured in 24-well plates for 24 h, then were incubated for 4 h in medium with PLN/C6 and APLN/C6 (equivalent concentration of C6 at 50 ng mL^{-1}), respectively. Thereafter, the cells were observed under an inverted fluorescence microscope (Eclipse Ts2-FL, Nikon Precision Inc., CA, USA) and analyzed with a flow cytometer (FACS Celesta, Becton, Dickinson and Company, NJ, USA).

The ration of APLN/MB uptake by BV-2 cells and SH-SY5Y cells

The ratio of APLN/MB uptake by BV-2 cells and SH-SY5Y cells were evaluated by establishing a Transwell cell model. The BV-2 cells (2×10^5 cells well^{-1}) were cultured in the upper chamber and the SH-SY5Y cells at a density of 2×10^5 cells well^{-1} were seeded in the lower chamber. APLN/C6 was pre-incubated with Aβ for 12 h to form the APLN/C6/Aβ nanoclusters. Thereafter, culture medium containing APLN/C6 or APLN/C6/Aβ (50 ng mL^{-1} of C6) was added into the Transwell (0.4 μm chambers, Corning Co, USA). After incubation for 4 h, the BV-2 cells and SH-SY5Y cells were rinsed by PBS and collected for flow cytometer analysis. To correct for any cellular auto-fluorescence, the fluorescence values of control cells incubated with unlabeled APLN or APLN/Aβ were subtracted from their respective values. The amount of cellular uptake by different cells was expressed as geometric mean fluorescence intensity (GMFI).[5] The uptake ratio (UR%) was calculated as follow: UR% = the GMFI of BV-2 cells / the GMFI of SH-SY5Y cells.

Cytotoxicity of APLN/MB/Aβ towards both BV-2 cells and SH-SY5Y cells

SH-SY5Y cells and BV-2 cells were seeded in 96-well plates at a density of 2×10^4 cells well^{-1}, respectively. To evaluate the cytotoxicity of APLN/MB after capturing Aβ oligomer (10 μmol L^{-1}), APLN/MB was pre-incubated with Aβ oligomer for 12 h, following by co-incubation with SH-SY5Y cells and BV-2 cells, respectively. Cells co-incubated with Aβ oligomer (10 μmol L^{-1}) or FBS-free culture medium were usded as control. After incubation for 24 h, the MTT solution was added to the plates and the absorbance was measured by a microplate reader at 490 nm. The cell viability was calculated by the following equation: cell viability (%) = O.D.sample /O.D.control × 100%.

In vitro blood-brain barrier model and penetration analysis

The hCMEC/D3 cells at a density of 6×10^5 cells cm^{-2} were cultured on the apical chamber of the Transwell insert (0.4 μm chambers, Corning Co, USA) and the cell-free chamber was

used as control. The BV-2 cells and the SH-SY5Y cells at a density of 2 × 10^5 cells cm^{-2} were seeded in the basal side of the lower chamber, respectively. The medium was replaced every 24 h. Trans-endothelial electrical resistance (TEER) values were measured every 24 h by a cell resistance meter. When the TEER value reached 50 Ω cm^2 and remained stable, the medium in the upper chamber was replaced with the medium containing PLN/C6 and APLN/C6 (equivalent concentration of C6 at 50 ng mL^{-1}).

After incubated for 4 h, the BV-2 cells and the SH-SY5Y cells in the lower chamber were fixed with paraformaldehyde for 20 min and dyed with DAPI (2 μg mL^{-1}) for 8 min. The cells were observed by an inverted fluorescence microscope.

In vivo and ex vivo imaging

The BBB penetration of nanocomposites in vivo was evaluated by the imaging system (VISQUE, Vieworks Co., Ltd., Gyeonggi-do, Korea). MB was replaced with near-infrared dye DiR, AD mice were intravenously injected with PLN/DiR (0.4 mg kg^{-1} of DiR) and different αAng modification ration (1%, 5%, 10% and 15%) of APLN/DiR (0.4 mg kg^{-1} of DiR), respectively (n = 3). The fluorescence images of AD mice were taken at 2, 4, 8, 12 and 24 h, while AD mice were anesthetized with 2% isoflurane. When finished imaging at 24 h, the AD mice were sacrificed and the brain and major organs were isolated for ex vivo imaging. The brains and organs were analyzed by Living Image Software.

Cellular uptake of $A\beta_{1-42}$

The BV-2 cells at a density of 2 × 10^5 cells $well^{-1}$ were cultured in a Poly-$_L$-Lysine pre-coated on 24-well plates for 12 h. To assess the cellular uptake of $A\beta_{1-42}$ by BV-2 cells in the presence of PLN/MB and APLN/MB, replacing medium with FBS-free medium containing FAM-$A\beta_{1-42}$ (2 μg mL^{-1}) respectively. Thereafter, PLN/MB and APLN/MB were added with the apoA-I concentration of 0.05, 0.1, 1, 5 and 10 μg mL^{-1} for 4 h, respectively. The cells incubated with FBS-free medium were used as control. The cells were collected for fluorescence activated cell sorting (FACS) assay after being rinsed by PBS.

Intracellular Aβ degradation assay

The BV-2 cells (2 × 10^5 cells $well^{-1}$) were cultured in 6-well plates for 24 h and incubated with $A\beta_{1-42}$ (2 μg mL^{-1}) with PLN/MB or APLN/MB at the apoA-I concentration of 0.05, 0.1, 1, 5, μg mL^{-1} for 4 h, respectively. Thereafter, the $A\beta_{1-42}$-containing culture medium were replaced by FBS-free medium for further incubation for 4 h. Then, the BV-2 cells were washed by PBS and lysed in 1% SDS. The total protein content of cell lysates was measured

using a bicinchoninic acid (BCA) protein assay kit (Jiangsu, China). The level of intracellular $A\beta_{1-42}$ was analyzed via an enzyme linked immunosorbent assay (ELISA) kit and normalized to total protein in the lysates.

Colocalization assay

The BV-2 cells (2×10^5 cells well^{-1}) were seeded on glass-bottom tissue culture dishes. The medium was replaced by an FBS-free medium with FAM-$A\beta_{1-42}$ (2 μg mL^{-1}) in the presence of PLN/MB and APLN/MB before being cultured overnight. Lysosomes were stained with Lyso Tracker-Red for 45 min and nuclei were dyed by Hoechst33258 for 15 min, respectively. The co-location of FAM-$A\beta_{1-42}$ and lysosomes were observed under a confocal microscope (CLSM, LSM700, Carl Zeiss AG, Oberkochen, Germany).

OA-induced phosphorylated Tau of neuron cells

The SH-SY5Y cells at a density of 2×10^4 cells well^{-1} were cultured in 96-well plates for 24 h. To induce phosphorylated Tau in cells, the medium was replaced by an FBS-free medium with the OA concentration varying from 10 nmol L^{-1} to 160 nmol L^{-1} and incubated for another 48 h. The SH-SY5Y cells cultured with medium only were used as control. Then, 100 μL of MTT solution (0.5%) was added and incubated for 4 h. After adding DMSO to replace the medium and incubated for 10 min, a microplate reader was use to measure the absorbance at 490 nm. The following equation calculated the viability of cells: cell viability (%) = O.D.sample /O.D.control × 100%.

MTT assay

The SH-SY5Y cells were incubated with OA (40 nmol L^{-1}) in the presence of MB, PLN/MB and APLN/MB at different MB concentrations (0.01, 0.1, 1, 2, 4 and 8 μg mL^{-1}) for another 48 h, respectively. The SH-SY5Y cells without MB-loaded preparations treatment were used as control. Thereafter, the medium was replaced by 100 μL of MTT solution (0.5%) and incubated for 4 h. A microplate reader was used to measure the absorbance of cells and the viability of cells was calculated by the following equation: cell viability (%) = O.D.sample /O.D.control × 100%.

Flow cytometry analysis of cell apoptosis

The SH-SY5Y cells (1×10^5 cells well^{-1}) were seeded onto 24-well plates and cultured for 12 h. Thereafter, cells were treated with OA (40 nmol L^{-1}) to induce the phosphorylated Tau. Meanwhile, cells were treated with free MB, PLN/MB and APLN/MB at MB concentration of 2 μg mL^{-1} for another 48 h, respectively. The SH-SY5Y cells without MB treatment were

used as control. Cells were resuspended and incubated with Annexin V-FITC/PI and propidium iodide for 10 min, followed by FACS analysis.

Thioflavin T (ThT) fluorescence assay

The SH-SY5Y cells (1×10^5 cells well^{-1}) were cultured in 24-well plates for 12 h and OA (40 nmol L^{-1}) was employed to induce the phosphorylated Tau for mimicking the Tau-induced cell damage. Thereafter, cells were treated with MB, PLN/MB and APLN/MB at MB concentration of 2 μg mL^{-1} for 48 h, respectively. After incubation, they were fixed with 500 μL of 4% paraformaldehyde for 20 min. Then, the samples were fixed with 4',6-diamidino-2-phenylindole (DAPI, 2 μg mL^{-1}) for 8 min and incubated in ThT (20 μmol L^{-1}) for 45 min. The photographs were taken and observed through an inverted fluorescence microscope.

Western blotting

The SH-SY5Y cells placed onto 6-well plates at a density of 2×10^5 cells well^{-1} were treated with OA (40 nmol L^{-1}) to induce Tau pathology. Meanwhile, MB, PLN/MB and APLN/MB, in which the concentration of MB was 2 μg mL^{-1}, were incubated for 48 h. Cells were homogenized in radio immunoprecipitation assay (RIPA) containing phenylmethylsulfonyl fluoride (PMSF), a protease inhibitor (1:100) and a phosphatase inhibitor (1:100) on ice. The total protein concentrations were determined by BCA assay and then the protein solution with 4 × loading buffer was boiled for 10 min. After being separated through a 10% SDS-page, the protein was transferred onto the poly vinylidene fluoride (PVDF) membrane. Then, the membranes were blocked with 5% BSA in tris buffered with tween-20 (TBST) for 1 h at room temperature, followed by incubating with primary antibodies at 4 ℃ overnight: Anti-pTau Ser396 antibody (1:10000, ab109390, Abcam), Anti-pAkt Ser473 antibody (1:2000, CST4060, Cell Signaling Technology), Anti-pGSK3β Ser9 antibody (1:1000, CST5558, Cell Signaling Technology), Anti-Akt antibody (1:1000, CST9272, Cell Signaling Technology), Anti-GSK3β antibody (1:1000, CST12456, Cell Signaling Technology), Anti-Tau antibody (1:1000, ab254256, Abcam) and Anti-β-actin (1:10000, GB11001, Servicebio). After being washed with TBST buffer, the membranes were incubated for 1 h with horseradish peroxidase (HRP)-linked secondary antibodies (goat anti-rabbit IgG H&L, rabbit anti-mouse IgG H&L, 1:10000, Servicebio) at room temperature and stained with enhanced chemiluminescence (ECL). The level of targeted proteins was photographed using a gel imaging system (Tanon 5200, Tanon Technology Co., Ltd., Shanghai, China).

Drug administration

The mice were randomly divided into six groups (*n* = 8). Sham and AD control groups were intravenously administrated with 200 μL of saline once three days for 4 weeks. PLN/MB and APLN/MB group mice were intravenously administrated 200 μL of PLN/MB and APLN/MB (equivalent dosage of MB at 2 mg kg^{-1}) once three days for 4 weeks, respectively. APLN group mice were intravenously administrated 200 μL of APLN at the equivalent lipid concentration with APLN/MB once three days for 4 weeks. MB p.o. group mice were intragastrically administrated with MB water solution at a MB dosage of 20 mg kg^{-1} once three days for 4 weeks.

Morris Water Maze (MWM) and nest construction task

The Morris Water Maze test was employed to monitor the learning ability and spatial memory of mice.[6] The whole process consisted of a 5 day training and a trial day on the sixth day. Mice would be put into a round pool, which was divided into 4 quadrants and remembered where the platform was by the symbol (square, circle, triangle and so on) on the wall during 5 training days. Before the training began, mice were transferred to the room for 2 h. The water temperature should be kept at (25 ± 1) ℃ and mice were dried after swimming. On the first 2 days, mice would be guided by a visible platform with a flag. Other 3 training days, the flag was removed and the platform was hidden under the water surface about 1 cm. Each mice was faced the wall of the pool to start swimming and the swimming track was recorded by a video-tracking system (DigBehv-MG, Shanghai Jiliang Software Technology Co., Ltd., Shanghai, China). If the mice were failed to reach the platform within 1 min, they were guided to the platform and remained for 10 s. All the mice were trained for thrice, which started from a random place (Northeast, Northwest, Southeast and Southwest) of pool in a training day. On day 6, each mouse was allowed to swim for 1 min without the platform. The latency, swimming pathway and times of crossing in the target quadrant of mice were recorded and analyzed by an equipped software for the MWM.

The nest construction experiment could evaluate the cognitive ability of mice. A mouse was put into a cage with a corncob padding of about 1 cm thick. Before the experiment began, pieces of square towel (5 cm × 5 cm) were placed into the cage to assess nest-constructing behavior.[7] After 24 h, the nest was captured and scored as follows: 0 = the towel was still square and no obvious biting; 1 = the towel was torn into some big pieces but no gathering; 2 = the towel was torn into some big pieces and was concentrated in the cage; 3 = the towel was torn into some small pieces and was on one side of cage; 4 = the towel was torn into some

small pieces and was gathered in a corner. The scores were graded blindly by more than three experimenters.

Western blotting for tissues

When finished testing, the Sham and AD mice were sacrificed. Brain tissues were collected and stored at -80 ℃. The brain tissue homogenate was prepared as follow: the equal quality of RIPA containing PMSF, a protease inhibitor (1:100) and a phosphatase inhibitor (1:100) was added, then brain tissue was grinded by a tissue grinder (TL2010S, DHS Biological Technology Co., Ltd., Tianjin, China) at 1800 rpm for 5 rounds (work 60 s per round and 10 s interval). After centrifugation at 12500 rpm, 4 ℃ for 20 min, the supernatant was collected. The protein in the supernatant was determined by BCA assay. The supernatant diluted with 4 × loading buffer was heated for 10 min and separated by 10% SDS-page. After electrophoresis, the protein was transferred to the PVDF membrane. Thereafter, the membrane was blocked by 5% BSA for 1 h at room temperature, incubated with the primary antibody as Anti-pTau Ser396 antibody (1:10000, ab109390, Abcam), Anti-pAKT Ser473 antibody (1:1000, CST4060, Cell Signaling Technology), Anti-pGSK 3β Ser9 antibody (1:2000, CST5558, Cell Signaling Technology), Anti-Akt antibody (1:1000, CST9272, Cell Signaling Technology), Anti-GSK3β antibody (1:1000, CST12456, Cell Signaling Technology), Anti-Tau antibody (1:1000, ab254256, Abcam) and Anti-β-actin (1:10000, GB11001, Servicebio) overnight at 4 °C and then with secondary antibody (goat anti-rabbit IgG H&L, rabbit anti-mouse IgG H&L, 1:10000, Servicebio) for another 1 h. Rinsing the membrane thrice with TBST after each liquid change. The blots were detected using an ECL reagent and analyzed by a gel imaging system.

Histology, immunofluorescence and immunohistochemical analysis

After behavioral tests, mice were euthanized and then perfused with paraformaldehyde. Brains and organs were isolated and embedded in paraffin. Brains and organs were fixed by submerging into 4% paraformaldehyde (PFA) in PBS, embedded into the paraffin, sectioned in coronal plane and processed. For histological analyses, brains and organs sections were stained with hematoxylin and eosin (H&E) and Nissl. For immunofluorescence analysis, antigen retrieval was performed (10 μmol L^{-1} citrate buffer, pH 6.0) for an efficient epitope exposure; endogenous peroxidase was quenched by treating the paraffin sections with 0.3% H_2O_2; and nonspecific binding of antibodies was eliminated using blocking buffer (1% BSA) for 1 h at room temperature. The primary antibody (anti-β-Amyloid 6E10, 803004, Biolegend) prepared in blocking buffer was applied for overnight at 4 ℃, followed by a

secondary antibody (Goat anti-mouse IgG Alexa Flour 488, GB25301, Servicebio) for 30 min incubation at room temperature.

For immunohistochemical analysis, antigen retrieval, endogenous peroxidase cancellation and nonspecific binding of antibodies blocking were performed according to the method mentioned above. The primary antibody (Anti-p-Tau Ser396, ab109390, Abcam) prepared in blocking buffer was applied for overnight at 4 ℃, followed by a secondary antibody for 30 min incubation at room temperature. For the secondary antibody and avidin-biotinylated peroxidase system, we used the HRP-Polymer anti-Mouse/Rabbit IHC Kit (MaxVision, 5010).

Safety profiles evaluation

To evaluate the safety of nanocomposites, hemolysis was investigated. After centrifugation at 1500 rpm, new rabbit red blood cells (RBC) were collected. PLN/MB and APLN/MB were diluted with saline at various concentrations and mixed with 2% RBCs suspension. Distilled water and saline mixed with 2% RBCs were used as positive controls ($A_{100\%}$) and negative ($A_{0\%}$), respectively. All the samples were incubated at 37 ℃ for 1 h. Thereafter, the suspension was collected by centrifugation at 3000 rpm for 10 min and detected by spectrophotometry at 570 nm. The percentage of hemolysis was calculated as follow: Hemolysis (%) = $(A_{sample} - A_{0\%})/(A_{100\%} - A_{0\%}) \times 100\%$.

For the bio-safety analysis, the mice were sacrificed and the major organs were collected for HE staining. Organs from the saline treated mice and AD mice were used as control.

Statistical Analysis

All the details about sample size, data presentation, statistical analysis and significant differences are provided in the figure captions. Unpaired two-tailed student's t-test was used for two-group comparison. One-way ANOVA, followed by Tukey post hoc analysis was used for multi-group comparison. The differences were displayed as significant for $*p < 0.05$, $**p < 0.01$ and $***p < 0.001$. Data were expressed as mean ± standard deviation (SD). Statistical analysis of data was performed by Prism GraphPad 9 software.

Table S1. Characterization of the PLN/MB and APLN/MB. (Mean ± SD, n = 3)

Samples	PDI	Size (nm)	Zeta potential (mV)	EE (%)	DL (%)
PLN/MB	0.239 ± 0.031	78.60 ± 0.34	-28.64 ± 0.31	70.53 ± 0.95	2.50 ± 0.07
APLN/MB	0.234 ± 0.002	96.90 ± 1.66	-29.87 ± 0.57	71.88 ± 1.30	2.44 ± 0.03

Table S2. The average affinity constants obtained from the binding interaction between PLN/MB and APLN/MB to $A\beta_{1-42}$ monomer and $A\beta_{1-42}$ oligomer, respectively. (Mean ± SD, n = 3)

Ligands	Parameter	PLN/MB	APLN/MB
	K_a ($M^{-1}s^{-1}$)	1.51×10^6	1.97×10^6
$A\beta_{1-42}$ monomer	K_d (s^{-1})	3.10×10^{-3}	1.99×10^{-3}
	K_D (M)	2.05×10^{-9}	1.01×10^{-9}
	K_a ($M^{-1}s^{-1}$)	4.90×10^5	5.39×10^5
$A\beta_{1-42}$ oligomer	K_d (s^{-1})	2.20×10^{-4}	1.50×10^{-4}
	K_D (M)	4.49×10^{-10}	2.78×10^{-10}

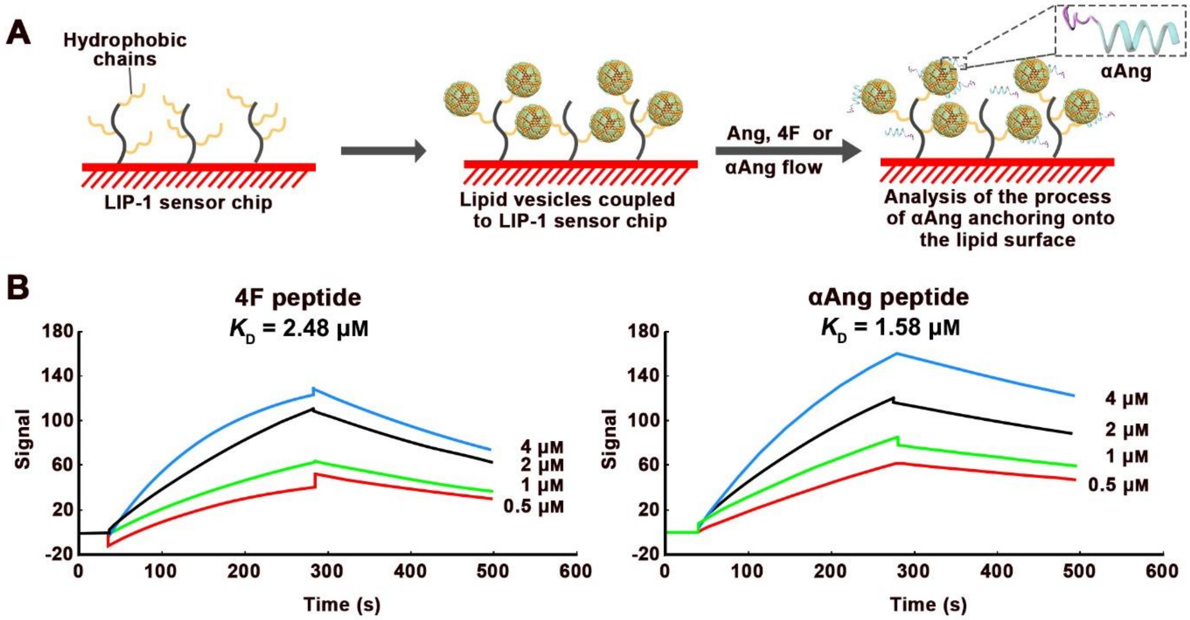

Figure S1. A) Schematic illustration of αAng anchoring onto the lipid surface by SPR. B) The binding affinity of 4F peptide and αAng to lipid nanoparticles was measured by SPR, respectively.

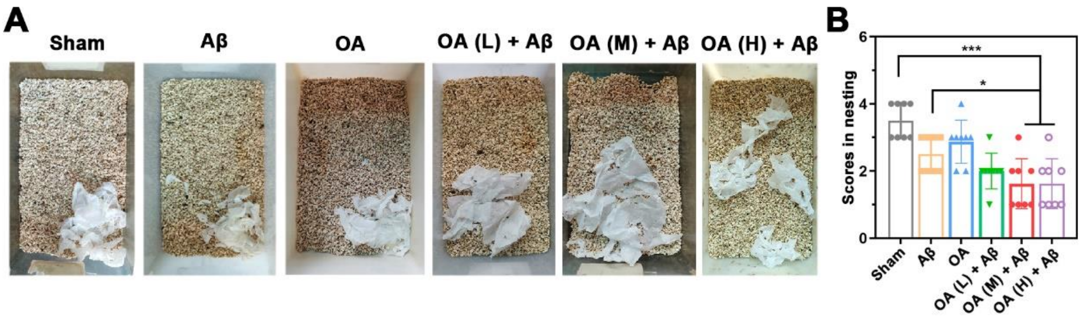

Figure S2. A) Representative images and B) scores of nest behavior for each group. Data were presented as mean ± SD (n = 8). *p < 0.05 and ***p < 0.001.

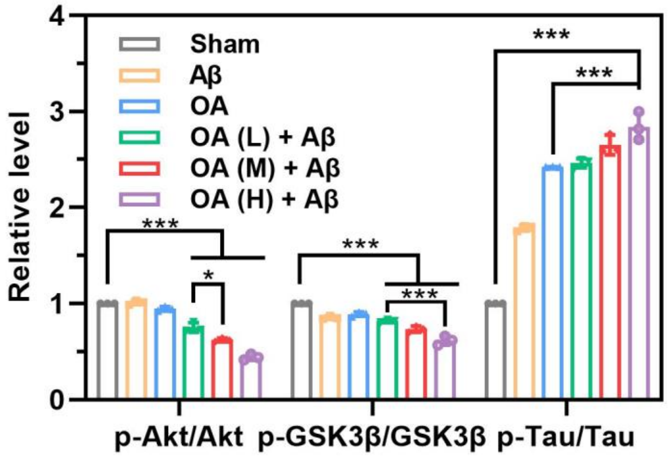

Figure S3. Quantification results of p-Akt, p-GSK3β and p-Tau in mice brain. p-Akt, p-GSK3β and p-Tau were normalized to total Akt, GSK3β and Tau, respectively. Data were presented as mean ± SD (n = 3). *p < 0.05 and *** p < 0.001.

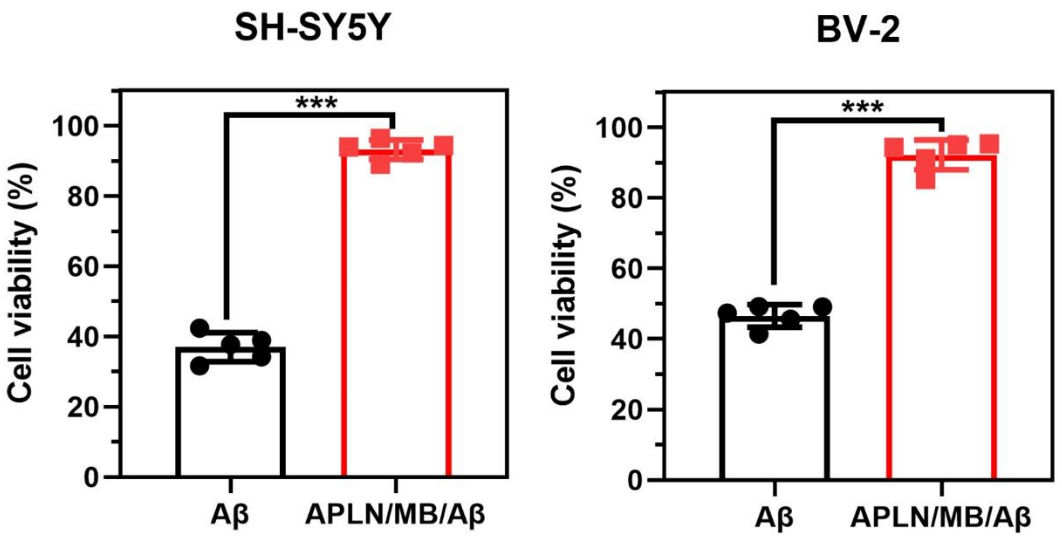

Figure S4. The cell viability of SH-SY5Y cells and BV-2 cells in the presence of Aβ and APLN/MB/Aβ, respectively (n = 5). Data were presented as the mean ± SD. *** $p < 0.001$.

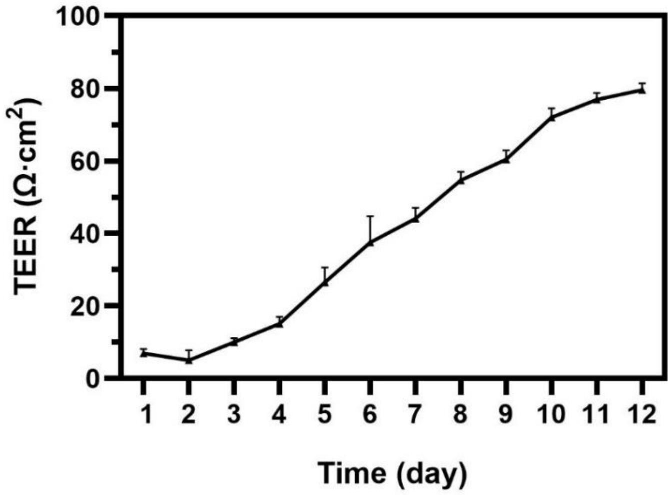

Figure S5. The growth curve of the hCMEC/D3 cell monolayer TEER value in 12 days. Data were presented as the mean ± SD (n = 5).

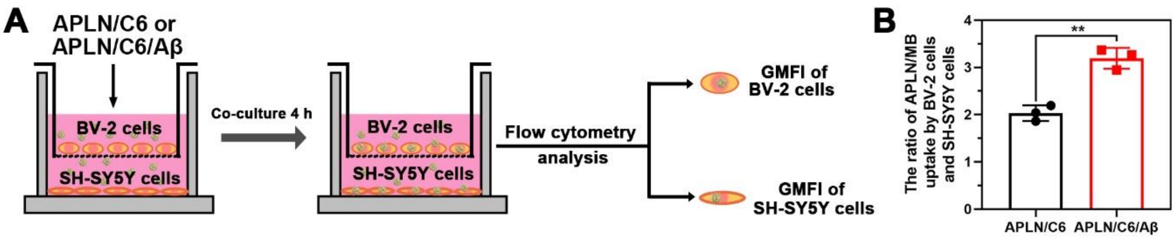

Figure S6. A) Schematic illustration of the BV-2 cells and SH-SY5Y cells coculture system for investigating the ratio of APLN/C6 and APLN/C6/Aβ uptake by microglia and neuronal cells. B) The ratio of APLN/C6 and APLN/C6/Aβ uptake by microglia and neuronal cells (n = 3). Data were presented as the mean ± SD. **$p < 0.01$.

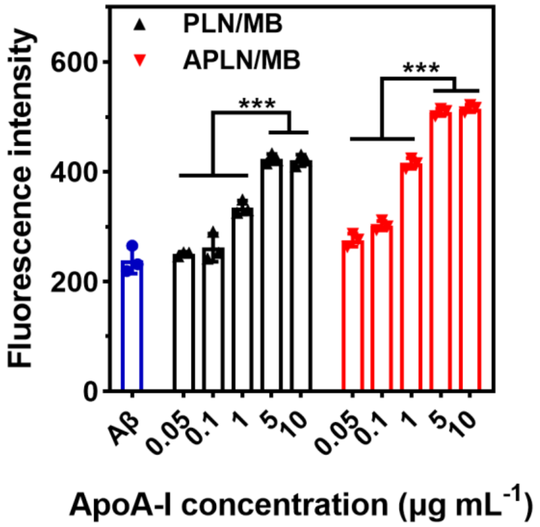

Figure S7. Cellular uptake of FAM-Aβ_{1-42} in microglia in the absence or presence of PLN/MB and APLN/MB, respectively (n = 3). Data were presented as the mean ± SD. ***p < 0.001.

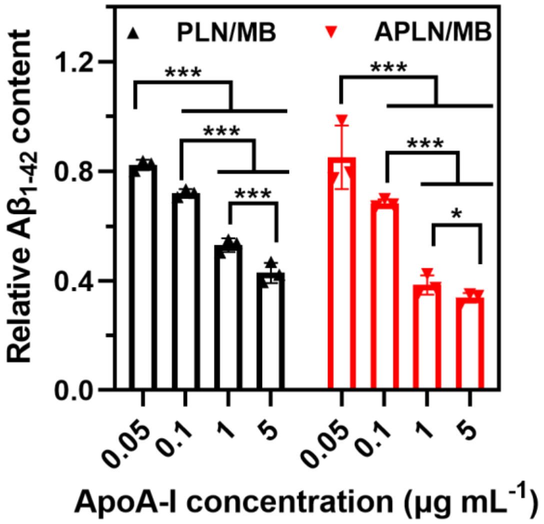

Figure S8. Relative intracellular Aβ levels quantified by ELISA after co-incubation with Aβ and APLN/MB or PLN/MB (n = 3). Data were presented as the mean ± SD. *p < 0.05 and ***p < 0.001.

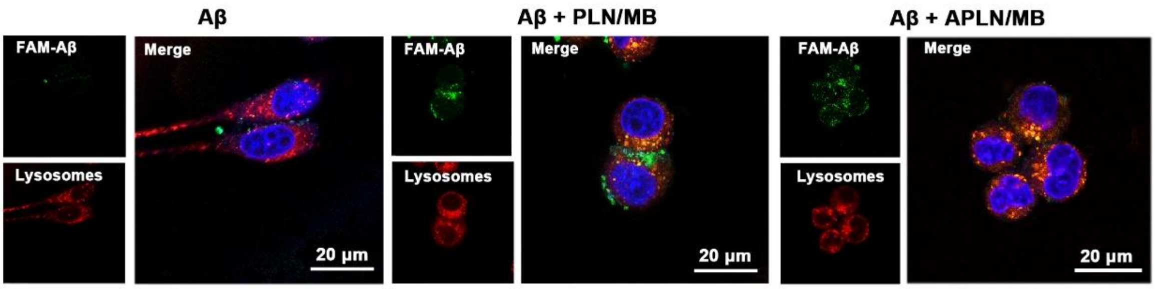

Figure S9. CLSM images for colocalization analysis of FAM-A$\beta_{1\text{-}42}$ with lysosomes of microglia with the absence or presence of APLN/MB or PLN/MB after incubation for 4 h. Scale bar = 20 μm.

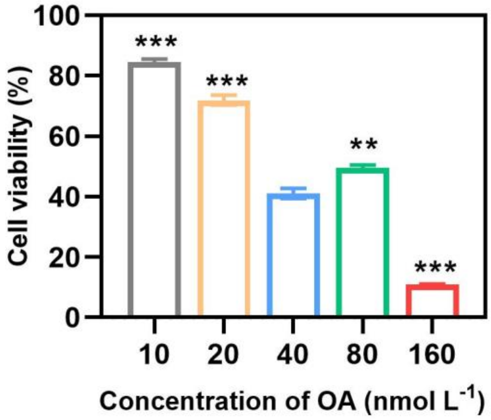

Figure S10. Cell viability of SH-SY5Y cells after 48 h of co-incubated with okadaic acid (OA). Data were presented as the mean ± SD (n = 5). with ** p < 0.01 and *** p < 0.001 compared to the OA (40 nmol L^{-1}) group.

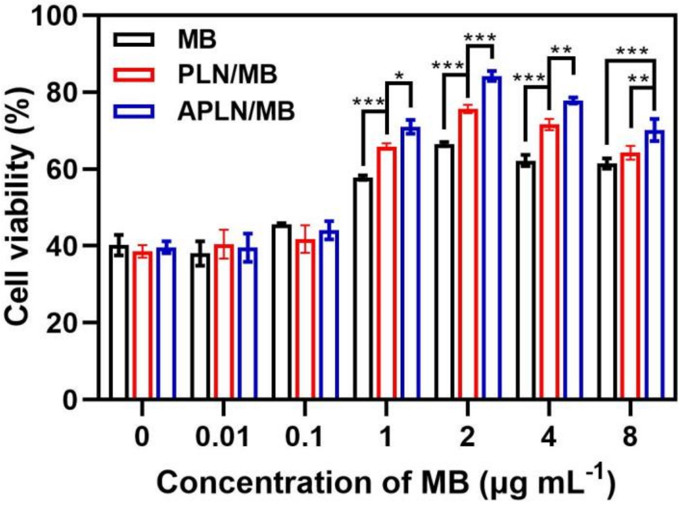

Figure S11. Protection effect of MB, PLN/MB and APLN/MB on OA (40 nmol L^{-1}) induced cytotoxicity of SH-SY5Y cells, respectively. Data were presented as the mean ± SD (n = 5). * $p < 0.05$, ** $p < 0.01$ and *** $p < 0.001$.

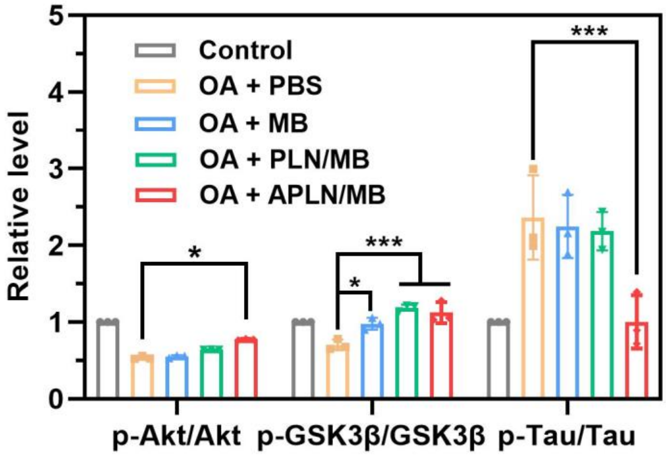

Figure S12. Quantification results of p-Akt, p-GSK3β and p-Tau in SH-SY5Y cells after treatment with MB, PLN/MB and APLN/MB. p-Akt, p-GSK3β and p-Tau were normalized to total Akt, GSK3β and Tau, respectively. Data were presented as mean ± SD (n = 3). * $p <$ 0.05 and *** $p < 0.001$.

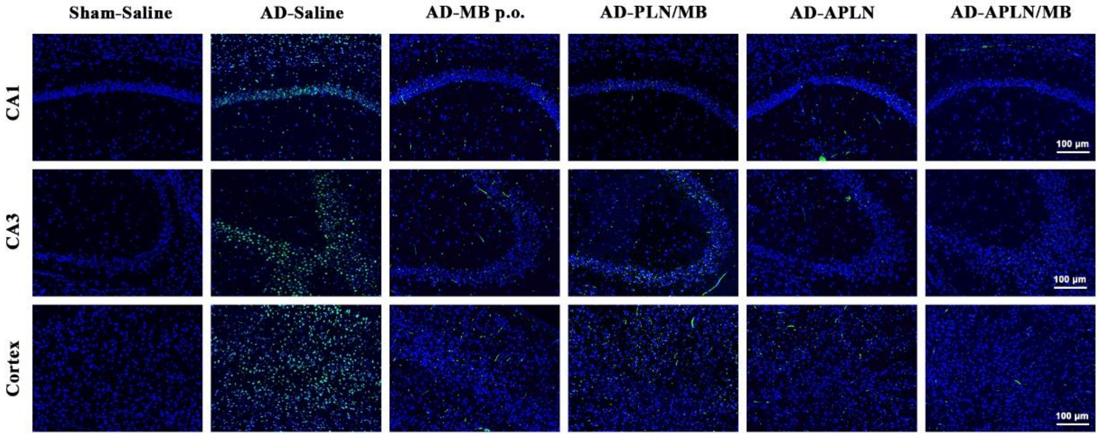

Figure S13. Immunostaining of Aβ deposition in the CA1, CA3 and cortex region of Sham mice or AD model mice treated with saline or different preparations for 4 weeks. Scale bar = 100 μm.

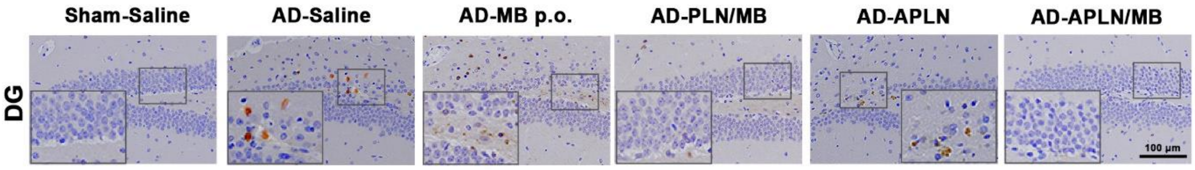

Figure S14. Representative images of NFTs in the DG region of Sham mice and AD model mice administered with saline and different formulations for 4 weeks. Scale bars = 100 μm.

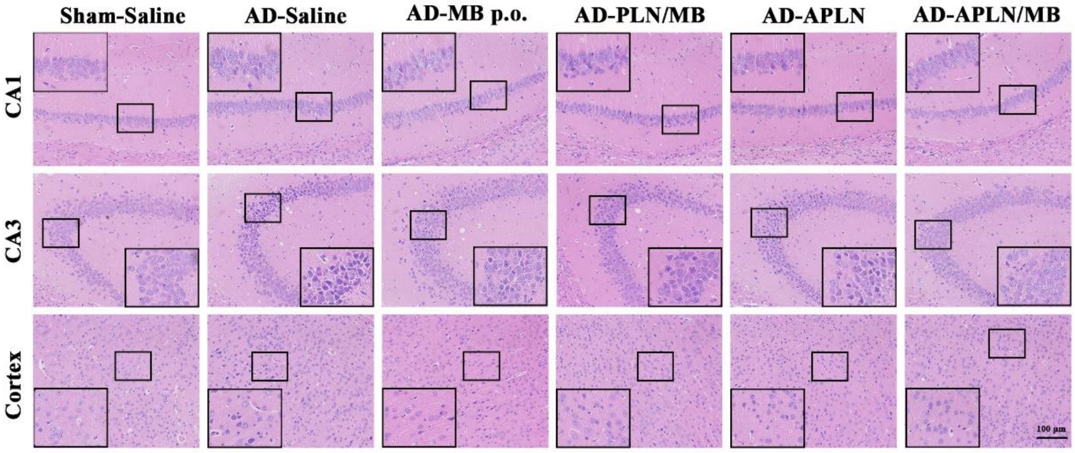

Figure S15. HE staining of neurons in the CA1, CA3 and cortex region of Sham mice or AD model mice treated with saline or different preparations for 4 weeks. Scale bar = 100 μm.

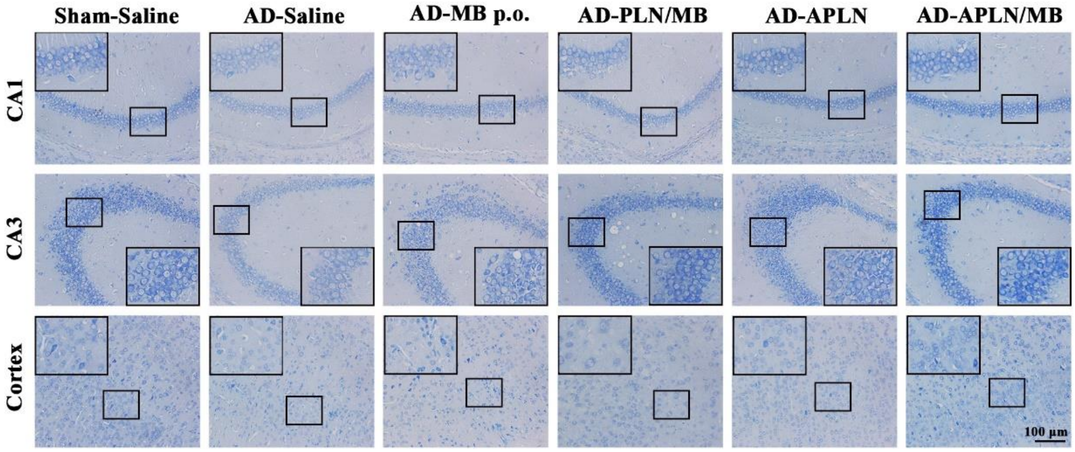

Figure S16. Nissl staining of neurons in the CA1, CA3 and cortex region of Sham mice or AD model mice treated with saline or different preparations for 4 weeks. Scale bar = 100 μm.

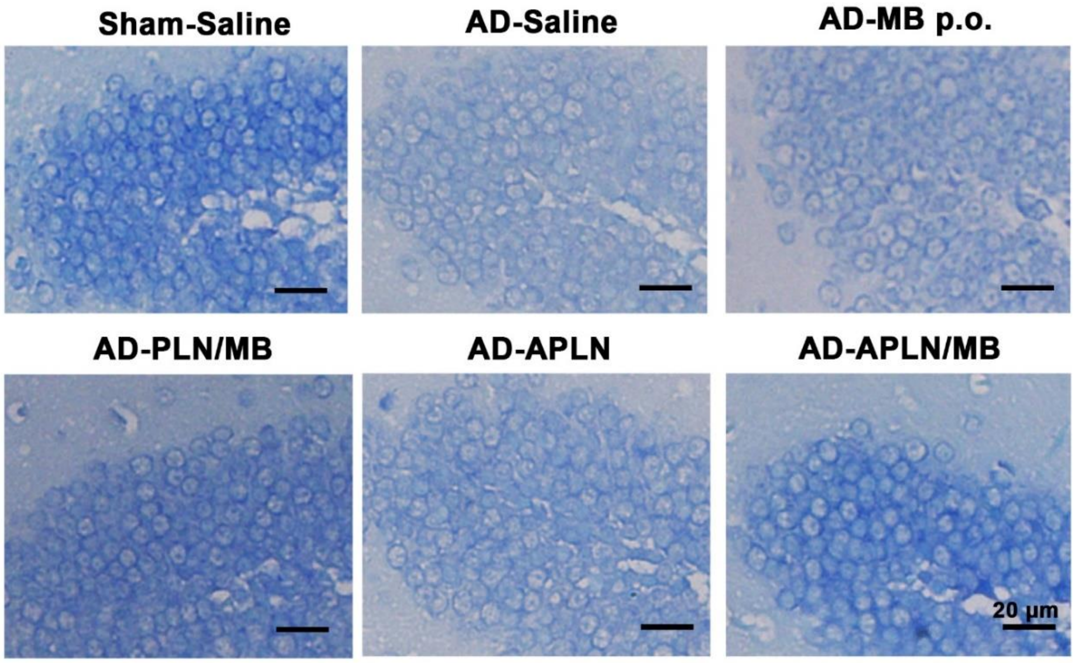

Figure S17. Enlarged Nissl imaging of neurons in the DG region of Sham mice or AD model mice treated with saline or different preparations for 4 weeks. Scale bar = 20 μm.

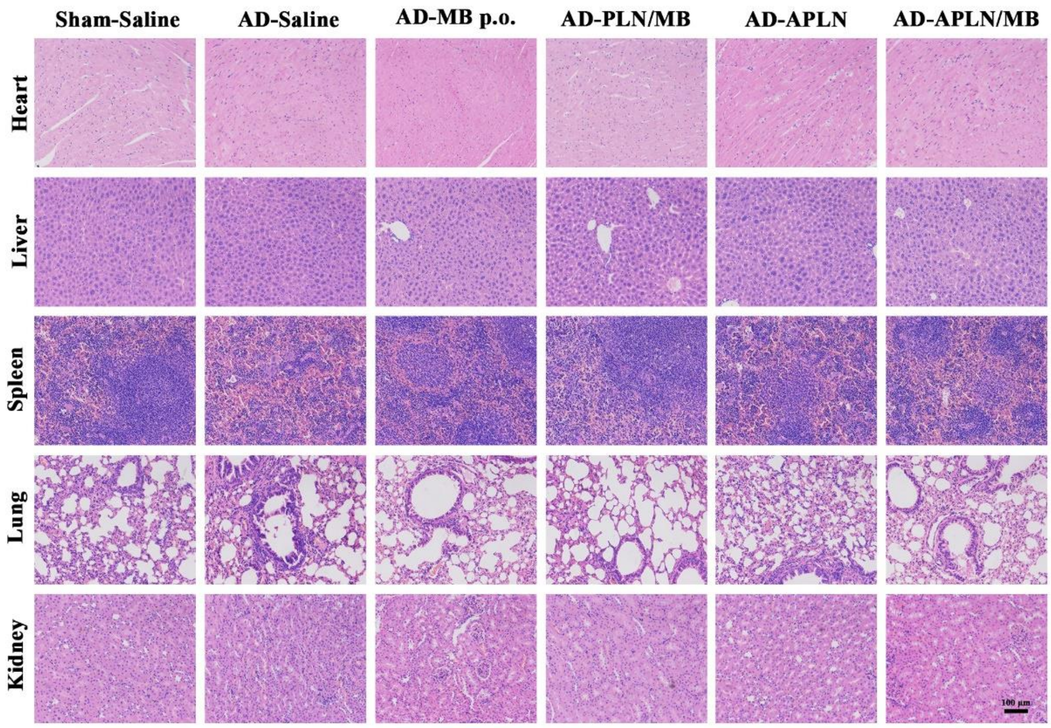

Figure S18. HE staining of the major organs of Sham mice or AD model mice treated with saline or different preparations for four weeks respectively. Scale bar = 100 μm.

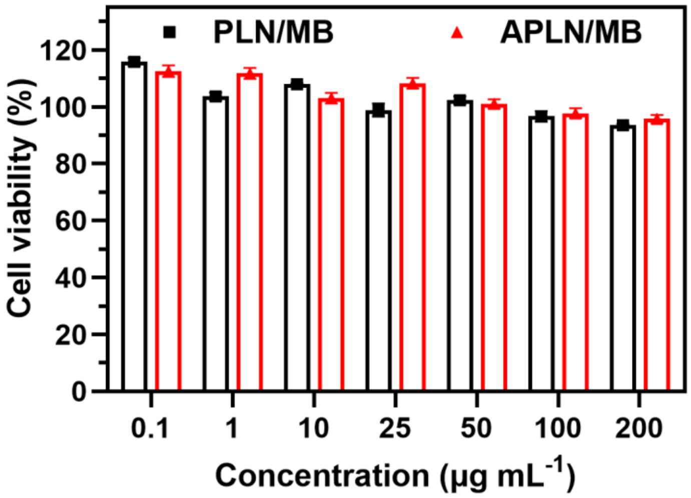

Figure S19. The cell viability of hCMEC/D3 cells treated with different concentrations of PLN/MB and APLN/MB for 24 h, respectively. Data were presented as the mean ± SD (n = 5).

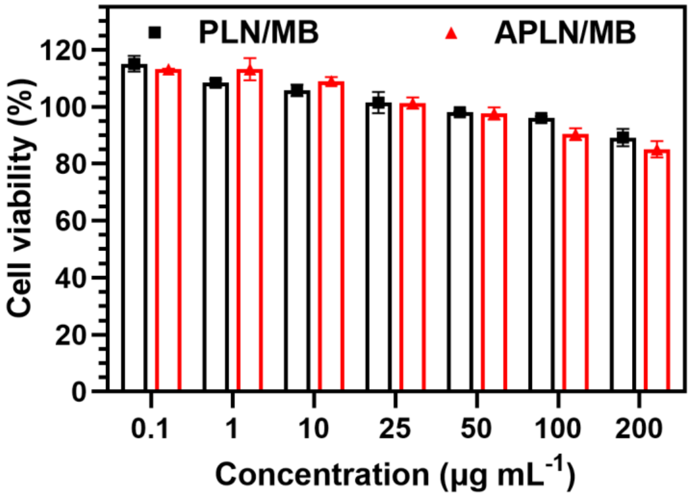

Figure S20. The cell viability of BV-2 cells treated with different concentrations of PLN/MB and APLN/MB for 24 h, respectively. Data were presented as the mean ± SD (n = 5).

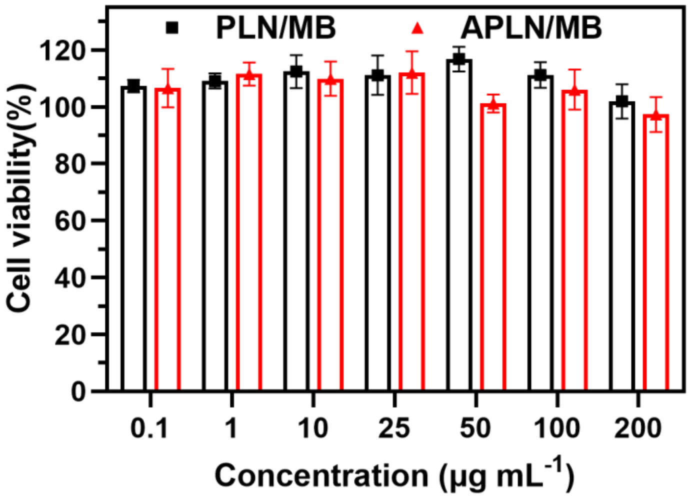

Figure S21. The cell viability of SH-SY5Y cells treated with different concentrations of PLN/MB and APLN/MB for 24 h, respectively. Data were presented as the mean ± SD (n = 5).

References

[1] S. Oddo, A. Caccamo, J. D. Shepherd, M. P. Murphy, T. E. Golde, R. Kayed, R. Metherate, M. P. Mattson, Y. Akbari, F. M. LaFerla, *Neuron* **2003**, *39*, 409.

[2] T. Pairojana, S. Phasuk, P. Suresh, S.-P. Huang, N. Pakaprot, S. Chompoopong, T.-C. Hsieh, I. Y. Liu, *Brain Res.* **2021**, *1762*, 147437.

[3] C. Zhang, X. Wan, X. Zheng, X. Shao, Q. Liu, Q. Zhang, Y. Qian, *Biomaterials* **2014**, *35*, 456.

[4] H. Wang, H. J. Sui, Y. Zheng, Y. B. Jiang, Y. J. Shi, J. Liang, L. Zhao, *NANOSCALE* **2019**, *11*, 7481.

[5] a) J. Youshia, M. E. Ali, V. Stein, A. Lamprecht, *Nanomedicine: Nanotechnology, Biology and Medicine* **2020**, *29*, 102244; b) M. Mao, Y. Xu, X. Y. Zhang, L. Yang, X. B. An, Y. Qu, Y. N. Chai, Y. R. Wang, T. T. Li, J. Ai, *J Neuroinflammation* **2020**, *17*, 244.

[6] M. A. Mifflin, W. Winslow, L. Surendra, S. Tallino, A. Vural, R. Velazquez, *Neurobiol. Aging* **2021**, *101*, 130.

[7] Y. Zhou, F. Zhu, Y. Liu, M. Zheng, Y. Wang, D. Zhang, Y. Anraku, Y. Zou, J. Li, H. Wu, X. Pang, W. Tao, O. Shimoni, A. I. Bush, X. Xue, B. Shi, *Sci Adv* **2020**, *6*, eabc7031.
